# Characterization of Two Self-Sufficient Monooxygenases, CYP102A15 and CYP102A170, as Long-Chain Fatty Acid Hydroxylases

**DOI:** 10.4014/jmb.1911.11048

**Published:** 2020-01-09

**Authors:** Hemraj Rimal, Woo-Haeng Lee, Ki-Hwa Kim, Hyun Park, Tae-Jin Oh

**Affiliations:** 1 Department of Life Science and Biochemical Engineering, Sunmoon University, Asan 3460, Republic of Korea; 2 Division of Biotechnology, College of Life Sciences and Biotechnology, Korea University, Seoul 0841, Republic of Korea; 3 Department of Pharmaceutical Engineering and Biotechnology, Sunmoon University, Asan 1460, Republic of Korea; 4 Genome-based BioIT Convergence Institute, Sunmoon University, Asan 3160, Republic of Korea

**Keywords:** *Bacillus* sp., cytochrome P450, fatty acid hydroxylation, *Paenibacillus* sp., self-sufficient monooxygenase

## Abstract

Self-sufficient P450s, due to their fused nature, are the most effective tools for electron transfer to activate C-H bonds. They catalyze the oxygenation of fatty acids at different omega positions. Here, two new, self-sufficient cytochrome P450s, named ‘CYP102A15 and CYP102A170,’ from polar *Bacillus* sp. PAMC 25034 and *Paenibacillus* sp. PAMC 22724, respectively, were cloned and expressed in *E. coli*. The genes are homologues of CYP102A1 from *Bacillus megaterium*. They catalyzed the hydroxylation of both saturated and unsaturated fatty acids ranging in length from C_12_–C_20_, with a moderately diverse profile compared to other members of the CYP102A subfamily. CYP102A15 exhibited the highest activity toward linoleic acid with K_m_ 15.3 μM, and CYP102A170 showed higher activity toward myristic acid with K_m_ 17.4 μM. CYP10A170 also hydroxylated the Eicosapentaenoic acid at ω-1 position only. Various kinetic parameters of both monooxygenases were also determined.

## Introduction

Cytochrome P450s are heme-thiolate proteins that exist in almost all biological kingdoms. They can catalyze a wide variety of reactions, including stereo-selective hydroxylation, epoxidation, organic peroxide isomerization, and N-, O-, and S-dealkylation [1]. The reaction mechanism involves the activation of molecular oxygen, followed by the introduction of a single oxygen atom into the organic substrate with the complementary reduction of other oxygen atoms to water [2]. To catalyze a variety of chemical reactions, most P450s interact with auxiliary proteins that are often known as redox partners [3]. Stereo- and regio-specific hydroxylation of non-activated hydrocarbons into industrially important compounds is accomplished by P450s, making them very important enzymes for biotechnology applications. These biotransformations are beneficial in the food, pharmaceutical, and cosmetics industries where high reaction acuteness of the complex substrate is essential [4]. Both microbial and mammalian P450s have been examined in the production of fine chemicals, fragrances, and pharmaceutical compounds as well as bioremediation [5].

Self-sufficient P450s are proteins with fused heme and reductase domains. The best example of this type of protein is CYP102A1 from *Bacillus megaterium*. The uniqueness of CYP102A1 lies in the fact that it can hydroxylate substrates much faster than eukaryotic P450 systems [6]. It is among the most studied microbial P450 monooxygenases, which catalyze the subterminal hydroxylation of medium- and long-chain fatty acids [7]. CYP102A1 can oxidize both saturated and unsaturated fatty acids, saturated fatty amides, and fatty alcohols [8-10]. N-acylated amino acids are also shown to be a superior substrate of CYP102A1 toward fatty acids [11]. The specific physiological implications of these reactions are unclear, but CYP102A1 is considered to be involved in fatty acid homeostasis and detoxification [12-14]. The function of CYP102A1 is to safeguard *B. megaterium* from polyunsaturated fatty acid (PUFA) toxicity, which is a form of “xenobiotic” metabolism [15]. Thus, the CYP102 family members are possible curative targets for organisms that must grow in the presence of PUFA [1]. Other natural fused P450 homologues of BM3 have been found, expressed, and analyzed as fatty acid hydroxylases in other diverse bacteria [16]. Slightly different fatty acid binding profiles and oxidation at different omega positions were observed in 102A series P450s [17]. CYP102D1 from *Streptomyces avermitilis* was shown to accept indole and anthracene as substrates in addition to fatty acids [18]. Similarly, CYP102B1 from *S. coelicolor* and 102H1 from *Nocardia farcinica* are 102 series P450s without fused heme and reductase domains [19, 20]. The discovery or engineering of enzymes that can catalyze reactions with higher selectivity and conversion rate will increase their value [21].

Here, we report the cloning, expression, and characterization of two new members of the CYP102A family. These monooxygenases were named CYP102A15 and CYP102A170 by the P450 nomenclature committee (http://drnelson.utmem.edu/CytochromeP450.html). The substrate preference of CYP102A15 from *Bacillus* and CYP102A170 from *Paenibacillus* species will help us better interpret the activity of CYP102 series P450s in host organism survival in lipid-rich environments. The data obtained show that CYP102A15 and CYP102A170 are unique compared to CYP102A1 (sequence identities ≤62%), which introduces diversity to the known class VIII monooxygenases. The substrate profile of both monooxygenases varies slightly from CYP102A1. Both CYP102A15 and CYP102A170 showed the highest activity for oxidation of long-chain unsaturated fatty acids with a carbon number between C_12_–C_20_.

## Materials and Methods

### Chemicals and Enzymes

All saturated and unsaturated fatty acids, 7-ethoxy coumarin, 7-hydroxy coumarin, coumarion, daidzein, and the derivatization reagent N, O-bis (trimethylsilyl) trifluoroacetamide (BSTFA) were purchased from Sigma-Aldrich (USA). All other chemicals and reagents of the highest grade were purchased from Tokyo Chemical Industry Co., Ltd. (Japan). Similarly, restriction enzymes, DNA ligase, and DNA polymerase were purchased from Takara (Japan).

### Sequence Alignment

The computer programs BLAST, FASTA, ClustalW, and GeneDoc were used to analyze and compare the nucleotide and amino acid sequences. Multiple sequence alignment of amino acid sequences was done using the ClustalW2 program, which uses a seeded guide tree and HMM profile-profile techniques. The program is available from the European Bioinformatics Institute website (http://www.ebi.ac.uk/clustalw). Alignments were visualized using the program Bioedit (http://www.mbio.ncsu.edu/BioEdit/bioedit.html). The amino acid sequence of CYP102A1 was used as query for a BLAST search to obtain the homologue sequences of these two enzymes.

### Phylogenetic Analysis

Phylogenetic analysis was conducted using MEGA version 6 [22] after sequence alignment and truncation to an equal length. Distances were maintained as a two-parameter model [23], and the clustering neighbor-joining method [24] was analyzed using bootstrap values based on 1000 replications [25].

### Bacterial Strains, Plasmids, and Growth Conditions

Basic information regarding the source, strains, and growth parameters related to *Bacillus* sp. PAMC 25034 and *Paenibacillus* sp. PAMC 22724 is given in the supplementary information (Table S1). Either Luria-Bertani (LB) agar plates or LB broth at 37°C were used to culture *Escherichia coli* strains. *E. coli* XL1-Blue MRF (Stratagene, USA) and *E. coli* BL21(DE3) were used for DNA manipulation and protein overexpression, respectively. Isopropyl-β-thiogalactopyranoside (IPTG; 0.4 mM) and 45 μl 5-bromo-4-chloro-3-indolyl-β-D-galactopyranoside (X-gal) were used for blue-white screening of transformants; ampicillin at 100 μg/ml was used as the selection marker. Similarly, pGEM-T Easy (Promega, USA) and pET28a (+) (Novagen, USA) were used as the cloning and overexpression vectors, respectively. Standard protocols were followed during all DNA manipulations, including ligation and enzyme digestion [26].

### Cloning and Expression of CYP102A15 and CYP102A170

Polymerase chain reaction (PCR) was performed in a thermocycler (Takara). The CYP102A15 gene was amplified using oligonucleotide primers; the forward primer was 5'- GGA TCC ATG CAA ACA TCA ATC -3' (BamHI), and the reverse primer was 5'- AGA TGT ATG GAG CTG ACT CGA G -3' (XhoI). Similarly, CYP102A170 was also amplified using the forward primer 5'- GGA TCC ATG GCG CAA ATT TCA GTT -3' (BamHI) and reverse primer 5'- AAG CTT CAC TCA GAT GCC TGT CC -3' (HindIII). Purified PCR products were ligated into T-vector for DNA amplification before transforming into *E. coli* XL1-blue. The sequences of the cloned CYP102A15 and CYP102A170 were confirmed using DNA sequencing. The verified clones were digested with the selected restriction enzymes. Ligation was repeated with plasmid pET28a(+); the product was transformed into *E. coli* BL21(C41). The *E. coli* transformants were spread on an LB plate containing an appropriate amount of kanamycin and incubated overnight at 37°C.

The confirmed clones of both monooxygenases were inoculated and grown in LB broth containing 50 μg/ml kanamycin. The temperature was maintained at 37°C until the cell density reached 0.6 at OD_600_. Similarly, 250 mM FeCl_3_ and 1 mM of 5-α-amino luvelunic acid (ALA) were added and the temperature was decreased to 20°C. After 15 min, 1 mM of IPTG was added and the sample was vigorously shaken at 200 rpm. After 30 h, the cell pellets were harvested and centrifuged at 3,500 ×g for 15 min and washed twice using ice cold Tris-HCl (50 mM) buffer (pH 7.4) containing 10% glycerol and 1 mM DTT. Finally, washed cell pellets were mixed with 1.5 ml of the respective buffer. An ultrasonicator at 35% power was used to lyse the cells, and centrifugation at 12,000 ×g for 25 min at 4°C was used to separate the soluble protein. The supernatant was bound using Talon Co^2+^-NTA resin (Clon Tech, USA) for Immobilized Metal Affinity Chromatography (IMAC). The binding resin was pre-equilibrated using equilibration buffer. The column loaded with resin-bound protein was washed with 7 bed volumes of 5 mM imidazole followed by elution with 5 bed volumes of 100 mM imidazole in potassium phosphate buffer (50 mM, pH 7.4) containing 100 mM potassium chloride. The His_6_-tagged eluted protein was concentrated using centricon; subsequently, the appropriate size was determined after analysis with 10% sodium dodecyl sulphate polyacrylamide gel electrophoresis (SDS-PAGE). All protein purification steps were performed at 4°C, and the concentrated protein was stored at -80°C until subsequent experiments.

### Spectroscopic and Kinetic Analysis of P450s

The UV-visible spectra of both P450s were recorded on a Shimadzu 1601PC spectrophotometer. The path length of the quartz cuvette used was 1 cm. CO reduction assays of CYP102A15 and CYP102A170 were conducted according to a published protocol [27]. In accordance with this method, the protein was diluted in 50 mM potassium phosphate buffer containing 10% glycerol. A small amount of sodium dithionite was added before dividing the dilution into two cuvettes. One cuvette was used as a reference, and the other was saturated with 50–60 bubbles of CO at a rate of 1 bubble per second. The dilution was scanned between 400 and 500 nm at room temperature on a Shimadzu 1601PC spectrophotometer. The CYP content was measured according to the difference in the absorbance values at 450 nm and 490 nm, and an extinction coefficient of 91 /mm/cm was used. The flavin concentration was analyzed after boiling the enzyme for 15 min in the dark. After centrifugation, the UV-visible spectra of the supernatant was recorded. The flavin concentration was calculated using an extinction coefficient of 9200 /M/cm at 473 nm [28].

The NADPH consumption assay was performed at 340 nm. The reaction was carried out using 250 μM NADPH, 200 μM fatty acid dissolved in dimethyl sulfoxide, and 0.5–1 μM enzyme. The total volume of the reaction was 250 μl. The obtained data were analyzed using GraphPad Prism software, and experiments were replicated at least three times.

### In Vitro Assay, Derivatization, and Analysis of the Hydroxylated Products

The in vitro P450 assays were performed using both saturated and unsaturated fatty acids as substrates along with some cyclic compounds. The reaction mixture consisted of 1 μmol purified protein, 5 mM MgCl_2_, 250 μM substrate, and 50 mM sodium phosphate buffer. The reaction was initiated by adding 500 μM NADPH before incubation at 30°C for 2.5 h. The reaction was stopped to perform an extraction with double volumes of diethyl ether after adding a pinch of anhydrous MgSO_4_. The extracted samples were dried under nitrogen gas or using a vacuum drier. Separate reactions were carried out using the recycling system to observe substrate depletion and avoid the accumulation of NADP that could inhibit the reaction. The dried sample was subjected to derivatization using BSTFA with 1% trimethylsilane and maintained at 75°C for 30 min before analyzing the derivatized fatty acids via GC-MS [29] on a GC (Agilent 6890N; USA) system, coupled to an Agilent 5973N electron impact mass selective detector equipped with a nonpolar capillary column (J&W MS-5, 30 m × 0.25 mm × 0.25 μm). A sample volume of 0.5 μl was injected for analysis. Helium was used as a carrier gas at a linear velocity of 30 cm/min. The column temperature was set at 80°C for 1 min, ramped to 210°C at a rate of 10°C/min, increased to 300°C at 40°C/min, and then held for 1 min for fatty acids with a chain length between C_7_–C_10_. Similarly, the column temperature was set at 150°C for 1 min, ramped to 260°C at a rate of 10°C/min, increased to 300°C at a rate of 40°C/min, and then held for 3 min for fatty acids with a chain length of C_11_–C_14_. For fatty acids with a chain length more than C_14_, the column temperature was set at 190°C for 1 min, increased to 300°C at a rate of 10°C/min, and then held for 5 min [30]. The hydroxylated products of fatty acids in silylated form were determined using their characteristic fragmentation patterns, as mentioned in previous publications [31-33].

### Nucleotide Sequence Accession Number

The nucleotide sequences used in this study were submitted to the NCBI GenBank database, with the accession numbers MH071450 and MH242621 for CYP102A15 and CYP102A170, respectively.

## Results and Discussion

### Phylogenetic Tree and Amino Acid Sequence Analysis

Both monooxygenases CYP102A15 and CYP102A170 encode amino acids similar to those encoded by CYP102A1 (BM3). The basic local alignment search tool (BLAST) was used to find enzymes like CYP102A15. We found that our protein has 55% identity with CYP102A1. Similarly, it showed a maximum resemblance of 62%with CYP102A3 from *B. subtilis*; however, the identity with CYP102D1 from *S. avermitilis* was only 38%. The sequence identities of CYP102A15 with other selected 102A subfamily members are presented in the supplementary data (Table S2). Similarly, CYP102A170 had 62% identity with CYP102A1 (BM3) and 40% identity with CYP102D1, which are both well-characterized P450s (Table S3).

We analyzed the phylogenetic relationship of the holoproteins to better understand the similarity among CYP102 enzymes of different bacterial strains. The phylogenetic tree showed that both our cytochromes clustered with CYP102A subfamily enzymes. However, there are different levels of sequence correlation within the CYP102A subfamily (Fig. S1). The holoproteins CYP102A1, A2, A3, A5, A7, A25, and A26, which are all from *Bacillus* strains, cluster together along with the enzyme from *S. pneumoniae*. CYP102A15 grouped more closely with other CYP102A holoenzymes from *B. pumilus*. Similarly, CYP102A170 was closer to CYP102A25 and CYP102A26 (Fig. S1).

The sequence alignment with the characterized CYP102 subfamily showed that our P450s contain a conserved AXEXGP-IF sequence in the opening of the substrate channel (Fig. 2A). Similarly, we found an EXXR motif in the I-helix, which has arginine as an important amino acid (Fig. 2B). We also found the exact heme-binding motif YKPFGNGQRACIG (Fig. 2B).

### Cloning, Expression, and Purification

Both monooxygenases were overexpressed in *E. coli* C41, and the protein amount was determined using CO reduction. The CO-reduced proteins showed a Soret peak at 450 nm; the absorbances at 450 nm and 490 nm were used to calculate the protein content. Protein yields of CYP102A15 and CYP102A170 were found to be 239 nmol and 330 nmol/l, respectively, which represent slightly less expression compared to type I P450s. The C-terminal His_6_-tag expressed protein analyzed by SDS-PAGE showed a band at approximately 120 kDa for CYP102A15 and 121 kDa for CYP102A170 (Fig. S2). A slightly alkaline pH of 7.8 could best purify the His_6_–tagged protein using metal affinity chromatography.

### Spectral Features

The oxidized forms of CYP102A15 and CYP102A170 showed Soret peaks of low spin at 420 nm, and the CO-reduced form showed an absorption maximum at 450 nm (Figs. 3A and 3B), which is typical for heme-binding proteins. α and β were observed at 535 and 567 nm for CYP102A15 and 537 and 569 nm for CYP102A170, respectively (data not shown). The incorporated flavin content of proteins was released by boiling for 15 min. It showed the presence of FMN and FAD via UV-visible spectrophotometry. The flavin-to-heme ratio was found to be 1.97 ± 0.01 and 1.93 ± 0.07 flavins per heme for CYP102A15 and CYP102A170, respectively. This indicated the complete incorporation of three cofactors in our P450s. The binding affinity of both saturated and unsaturated fatty acids to CYP102A15 and CYP102A170 cause a shift of the heme Soret peak from low spin at 420 nm toward high spin at 390 nm. A similar shift from 419 to 389 nm occurred for CYP102A170. The binding affinity was evaluated by spectrometrically analyzing the enzyme binding of each substrate.

It has been shown that all identified CYP102 subfamily self-sufficient P450s act on fatty acids (or other anionic fatty acyl incorporated compounds); however, they vary in their exact choice within the substrate family, with particular variability in fatty acid substrate chain length [8-10,16,17,34-36]. At the beginning, substrates were selected as fatty acids with different chain lengths and the unsaturation extent was tested for enzyme binding capacity. Substrate binding was observed by alternating from low-spin heme iron with a Soret band maximum at 419 nm in the UV-visible spectrum to high-spin heme iron with a Soret band maximum at 389 nm (Figs. 4, S3, and S4). Interestingly, CYP102A170 showed a low spin Soret band at 430 nm and a high spin Soret band at 410 nm with lauric acid and myristic acid (data not shown); however, it showed normal behaviour with other substrates. Not all substrates showed complete alteration from a low spin to higher spin state. However, partial spin change can be observed to determine the spectral dissociation constant (Table 1). Both our enzymes preferred saturated fatty acids with a chain length of C_12_–C_20_. We found that binding rates of CYP102A15 with lauric acid were almost two times stronger than that with margaric acid. CYP102A170 showed the strongest binding rate with margaric acid. In the case of unsaturated fatty acids, we found that CYP102A15 had a stronger binding rate with linoleic acid (C_18:2_) (13.95 μM) than with oleic acid (C_18:1_) (25.72 μM) or arachidonic acid (C_18:4_) (29.82 μM). CYP102A170 showed better binding rates with eicosapentaenoic acid (C_20:1_) (1.30 ± 0.08 μM); however, CYP102A170 exhibited better overall substrate affinity than CYP102A15 (Table 1).

### Catalytic Activity

Saturated fatty acids, unsaturated fatty acids and other cyclic compounds were the three main types of substrates used to determine the NADPH oxidation rate. We found that both CYP102A15 and CYP102A170 favored saturated and unsaturated fatty acids over cyclic compounds. They consumed NADPH from carbon number C_12_ to C_20_. CYP102A15 showed a better turnover number with myristic acid (81.67 nmol/nmol-CYP/min) than other fatty acids. In contrast, CYP102A170 exhibited an NADPH consumption rate of only 8.2 nmol/nmol-CYP/min with margaric acid. Both P450s demonstrated better NADPH consumption with unsaturated fatty acids, compared to saturated fatty acids (Fig. 5). Besides fatty acids, only 7-ethoxycoumarin consumed the NADPH (data not shown). Our cytochromes showed very little turnover of cyclic compounds; this was probably due to the small active site or greater hydrophilicity for acceptance of bigger cyclic compounds [37]. The coupling efficiencies, product formation rates (PFR), and Michaelis–Menten constants (K_m_) of CYP102A15 and CYP102A170 were also investigated. Here, coupling efficiency of CYP102A15 and CYP102A170 with lauric acid had the highest values, 90% and 72%, respectively. K_m_ with linoleic acid was 15.30 ± 4.70 μM for CYP102A15, and K_m_ with lauric acid was 13.37 ± 1.30 μM for CYP102A170 (Table 2).

### Reaction Product Identification

**Fig. 1:**
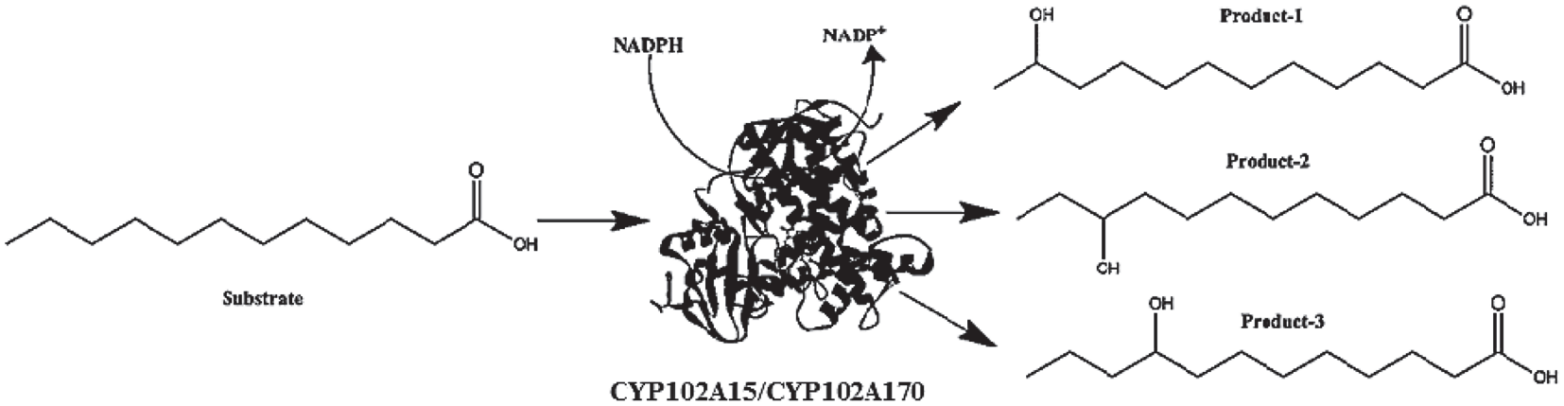
Schematic figure for hydroxylation of fatty acids using CYP102A15 and CYP102A170.

**Fig. 2:**
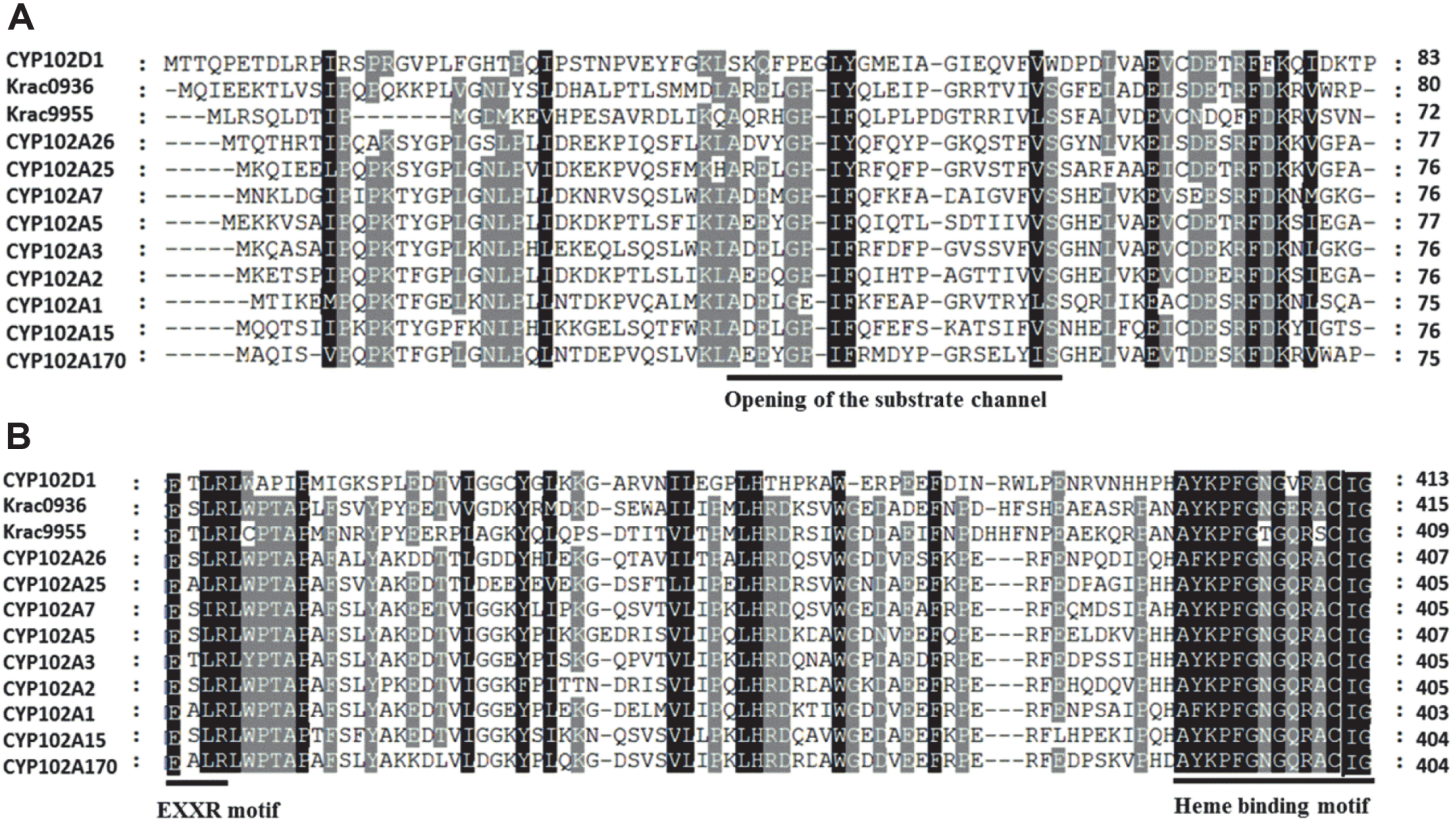
Relative data and amino acids sequence alignment of selected portions of CYP102A15 and CYP102A170 with those of other CYP102A subfamily members. (**A**) Critical amino acids in conserved regions of the substrate entrance channel. (**B**) EXXR motif in K-helix and heme-binding motif.

**Fig. 3:**
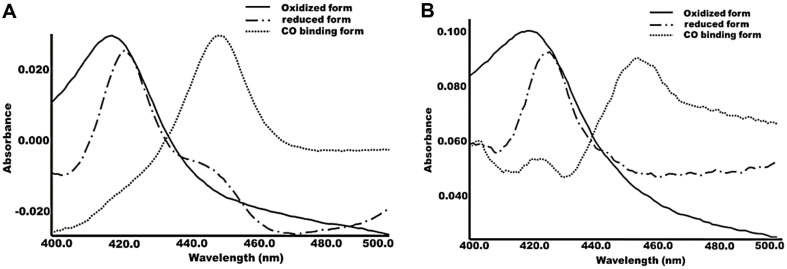
UV-visible absorption spectra of expressed CYP102A15 (**A**) and CYP102A170 (**B**). Solid line, oxidized form; dashed line, sodium dithionate-reduced form; and dotted line, CO-bound form.

**Fig. 4:**
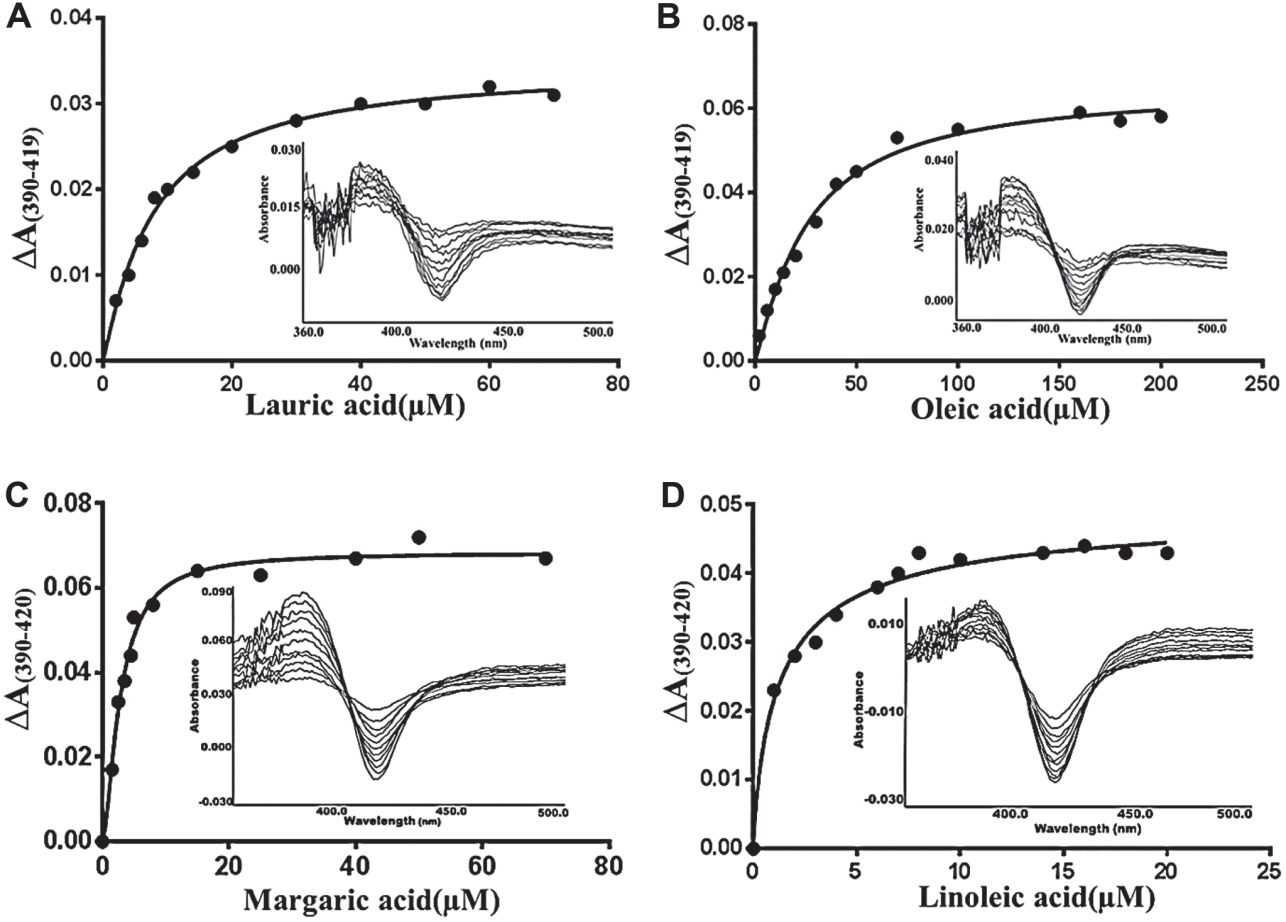
The titration of CYP102A15 and CYP102A170 with representative substrates. (**A**) The plot of absorbance change ΔA (A_389_–A_419_) vs. concentration of lauric acid. Inset: difference spectra of CYP102A15 with lauric acid. The CYP102A15 concentration was 2 μM. (**B**) The plot of absorbance change vs. oleic acid concentration. Inset: difference spectra of CYP102A15 with oleic acid. The CYP102A15 concentration used was 3 μM. The shift had a maximum peak at 389 nm and a minimum at 419 nm. (**C**) The plot of absorbance change ΔA (A_390_–A_420_) vs. concentration of the margaric acid. Inset: difference spectra of CYP102A170 with margaric acid. (**D**) The plot of absorbance change ΔA (A_390_–A_420_) vs. linoleic acid concentration. Inset: difference spectra of CYP102A170 with margaric acid. The CYP102A170 concentration was 3.5 μM.

**Fig. 5:**
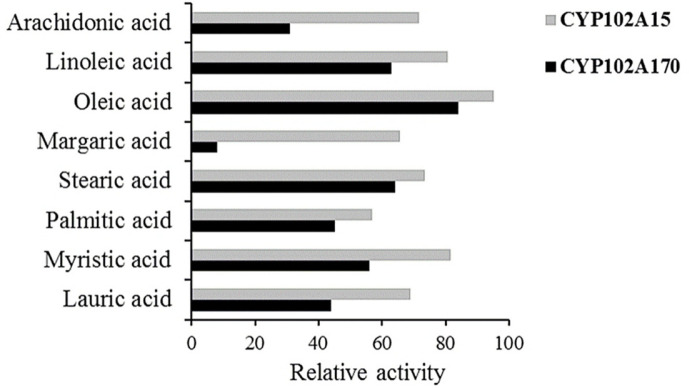
Comparison of relative NADPH oxidation rates between CYP102A15 and CYP102A170 against saturated and unsaturated fatty acids. NADPH oxidation rates were measured in nmol NADPH/min/nmol of CYP102A15 or CYP102A170.

CYP102A15 and CYP102A170 yielded a variety of omega fatty acid hydroxyl products. All the fatty acid products were analyzed using the specific GC-MS fragmentation pattern of trimethylsilylated hydroxy fatty acids (Fig. 1 and Table S4). They favored ω-1 over the ω-2 and ω-3 positions of saturated fatty acids. Most of the products were from the ω-1 to ω-3 position, and long-chain fatty acids after carbon chain number 18 showed the ω-1 position only (and Fig. S19). In the case of unsaturated fatty acids, most of the products were at the ω-1 and ω-2 positions (Fig. S4-S19 and Table S1).

In conclusion, CYP102A15 and CYP102A170 from *Bacillus* and *Paenibacillus* species were cloned and overexpressed in *E. coli* C41 and purified using metal affinity chromatography. Both are self-sufficient, fatty acid oxidizing P450s that have similarities to previously characterized P450s such A1, A2, A3, A5, A7, D1, Krac0936, Krac9955, A25, and A26. The in vitro study of enzyme activity showed that CYP102A15 and CYP102A170 have some differences in substrate preference between saturated and unsaturated fatty acids. Moreover, they can catalyze both even and odd chains. The length range of fatty acid oxidation was from C_12_–C_18_ for saturated fatty acids, and the maximum carbon chain length was 20 in the case of unsaturated fatty acids. They also exhibited oxidation at ω-1 and ω-2 as favorable positions; ω-3 was the least favorable position. Hydroxylation of eicosapentaenoic acid by CYP102A170 at only the ω-1 position highlighted the regio-selectivity of this enzyme. Further investigations related to mutagenesis and direct evolution of the P450 monooxygenase are underway. CYP102A family enzymes have often been used for protein engineering because they are self-sufficient, fast, and easy to handle. However, the substrates for wild-type CYP102A enzymes are mostly fatty acids. CYP102A15 has a broader substrate range than the CYP102A family enzymes as it catalyzes the oxidation of bulky cyclic compounds. We found that cyclic compounds such as 7-ethoxycoumarin indicate the oxidation capacities of CYP102A15; however, other bulky compounds could be their oxidation targets. These new fascinating self-sufficient enzymes could overcome limitations such as specific regio-selectivity or narrow substrate specificity. Furthermore, hydroxylation of eicosapentaenoic acid by CYP102A170 could add diversity to the CYP102A subfamily. Further research will focus on protein engineering and structural analysis based on comparative structure/function studies and screening substrates that can be converted into more valuable molecules by CYP102A15 and CYP102A170. Further investigations with branched, polyunsaturated, and long fatty acids, longer than 22 carbons in chain length, could improve the current understanding of the regio- and stereo-selectivity of the enzymes. To our knowledge, this is the first report of the hydroxylation of eicosapentaenoic acid by CYP102A170.

## Supplementary Data

Supplementary data for this paper are available on-line only at http://jmb.or.kr.

## Figures and Tables

**Table 1:** Binding parameters of fatty acids and hydroxylation at different omega (ω) positions by CYP102A15 and CYP102A170.

Fatty acids type	Substrates	CYP102A15		CYP102A170	
	
		K_d_ (μM)	Hydroxylation position	K_d_ (μM)	Hydroxylation position
Saturated	Lauric acid (C12)	7.78 ± 0.70	ω-1, ω-2, ω-3	6.46 ± 0.30	ω-1, ω-2, ω-3
Saturated	Myristic acid (C14)	14.94 ± 0.68	ω-1, ω-2	9.89 ± 0.39	ω-1, ω-2
Saturated	Palmitic acid (C16)	11.03 ± 1.30	ω-1, ω-2	5.01 ± 0.23	ω-1, ω-2, ω-3
Saturated	Stearic acid (C18)	14.94 ± 0.68	ω-1, ω-2	3.37 ± 0.20	ω-2, ω-3
Saturated	Margaric acid (C17)	17.93 ± 1.21	ω-1, ω-2, ω-3	2.83 ± 0.17	ω-1, ω-2
Unsaturated	Oleic acid (C18)	25.72 ± 2.80	ω-1, ω-2, ω-3	2.23 ± 0.09	ω-1, ω-2
Unsaturated	Linoleic acid (C18)	13.95 ± 1.50	ω-1, ω-2	1.36 ± 0.23	ω-1, ω-2
Unsaturated	Eicosapentaenoic acid (C20)	18.79 ± 3.80	-	1.30 ± 0.08	ω-1
Unsaturated	Arachidonic acid(C20)	29.82 ± 1.80	ω-1		

The dissociation constant (K_d_) was estimated using a single-binding model for spectral change ΔA (A_389_–A_419_) or ΔA (A_390_–A_420_) with increasing substrate concentrations added to 1 μM of protein until substrate saturation was acquired.

ND, not determined.

**Table 2:** Kinetic parameters of CYP102A15 and CYP102A170. Product formation rate was given in (nmol/ nmol-P450/min).

Substrates	CYP102A15			CYP102A170		

	K_m_ (μM)	PFR	CE (%)	K_m_ (μM)	PFR	CE (%)
Lauric acid	21.70 ± 3.60	7.00 ± 0.10	90	13.37 ± 1.30	31.68 ± 0.70	72
Myristic acid	386.10 ± 39.20	35.91 ± 1.20	80	17.43 ± 1.80	37.52 ± 1.90	67
Oleic acid	39.10 ± 6.90	23.10 ± 5.30	85	430.00 ± 98.00	41.50 ± 1.30	51
Linoleic acid	15.30 ± 4.70	2.70 ± 0.30	68	79.00 ± 7.20	37.17 ± 2.90	60

PFR=Product formation Rate, K_m_=Michalis Menten constant, CE=coupling efficiency
