# Supplementary material for: Characterization of Two Self-Sufficient Monooxygenases, CYP102A15 and CYP102A170, as Long-Chain Fatty Acid Hydroxylases
Source: J Microbiol Biotechnol. 2020 Jan 9;30(5):777–84. doi: 10.4014/jmb.1911.11048 (PMC9728198; doi:10.4014/jmb.1911.11048)

J. Microbial. Biotechnol.

10.4014/jmb.1911.11048

Table S1. Basic information related to CYP102A15 and CYP102A170. Both of the strains were grown in Reasoner's 2A (R2A) media at 15℃ and pH 7.2.

Name of cytochrome P450	CYP102A15	CYP102A170
GenBank accession number	MH071450	MH242621
Total number of amino acids	1,047	1,057
Tentative molecular weight	119 kDa	121 kDa
Organism	*Bacillus* sp. PAMC 25034	*Paenibacillus* sp. PAMC 22724
Source	Kara sea water	Kara sea sediment

Table S2. The sequence identities of the CYP102A15 from *Bacillus* sp. with privileged other CYP102A subfamily representative including CYP102A1 (*B. megaterium*), CYP102A2 (*B. subtilis*), CYP102A3 (*B. subtilis*), CYP102A5 (*B. cereus*), CYP102A7 (*B. licheniformis*), CYP102D1 (*Streptomyces avermitilis*), Krac9955 (*Ktedonobacter racemifer* DSM44963), and Krac0936 (*Ktedonobacter racemifer* DSM44963). Similarly, CYP102 members from *B. pumilus*, *B. safensis*, *B.* sp. AM13, *B. aerophilus*, *B. stratosphericus*, *B. xiamenensis*, *B. mojavensis*, *B. halotolerans*, and *B. malacitensis* were also included. Sequences and nomenclature were achieved either from the NCBI database or P450 homepage (http://drnelson.uthsc.edu/CytochromeP450.html).

CYP102 subfamily	Accession number	Identities (%)	Positives (%)	Gaps (%)	Score (bits)	Length (aa)
B. pumilus	WP_050944903.1	99	99	0	2,169 (5,620)	1,047
B. safensis	WP_046311196.1	91	95	0	2,006 (5,197)	1,047
B. sp. AM 13	WP_059375410.1	91	95	0	1,993 (5,164)	1,047
B. aerophilus	WP_041507223.1	89	95	0	1,979 (5,126)	1,047
B. stratosphericus	WP_052320288.1	89	94	0	1,973 (5,112)	1,047
S. pneumoniae	CVM94475.1	89	94	0	1,968 (5,099)	1,047
B. xiamenensis	WP_008357863.1	87	92	0	1,922 (4,980)	1,047
B. mojavensis	WP_024122280.1	63	77	0	1,389 (3,595)	1,054
B. halotolerans	WP_059335492.1	63	77	0	1,389 (3,594)	1,054
B. malacitensis	WP_059293535.1	62	77	0	1,387 (3,589)	1,054
CYP102A3 BSn5	WP_015714418.1	62	76	0	1,373 (3,553)	1,054
CYP102A1	P14779	55	72	0	1,220 (3,156)	1,049
CYP102A2	O08394	56	71	1	1,214 (3,140)	1,061
CYP102A3	O08336	62	75	0	1,363 (3,529)	1,054
CYP102A5	ADL27534	56	71	1	1,187 (3,072)	1,034
CYP102A7	WP_003183869	60	75	1	1,328 (3,437)	1,074
CYP102D1	WP_010982013	39	56	2	754 (1,947)	1,073
Krac9955	EFH88481	41	60	3	821 (2,121)	1,080
Krac0936	EFH80345	47	65	2	993 (2,566)	1,074

Table S3. The sequence identities of the CYP102A170 from *Paenibacillus* sp. with privileged other CYP102A subfamily representative including CYP102A1 (BM3).

CYP102 subfamily	Accession number	Identities (%)	Positives (%)	Gaps (%)	Score (bits)	Length (aa)
P. sp. AD87	WP_064638912.1	99	99	0	2,181 (5,651)	1,057
P. taichungensis	WP_094939341.1	98	98	0	2,150 (5,571)	1,057
P. pabuli	WP_062324544.1	95	97	0	2,078 (5,383)	1,057
P. amylolyticus	WP_076249610.1	93	96	0	2,043 (5,293)	1,057
P. campinasensis	WP_095263755.1	91	96	0	2,001 (5,185)	1,057
C. abortus	SHE14180.1	70	83	0	1,583 (4,099)	1,057
B. laterosporus	WP_026315237.1	68	83	0	1,557 (4,032)	1,059
A. aneurinilyticus	WP_021622834.1	69	83	0	1,554 (4,024)	1,058
B. laterosporus	WP_022586422.1	68	82	0	1,548 (4,007)	1,062
P. polymyxa	WP_025365419.1	68	81	0	1,520 (3,935)	1,058
B. sp. J13	WP_028404591.1	68	79	1	1,518 (3,931)	1,059
CYP102A1	P14779	62	76	0	1,359 (3,518)	1,049
CYP102A2	O08394	64	78	0	1,434 (3,713)	1,061
CYP102A3	O08336	61	75	0	1,360 (3,519)	1,054
CYP102A5	ADL27534	65	79	0	1,414 (3,659)	1,034
CYP102A7	WP_003183869	62	76	0	1,365 (3,532)	1,074
CYP102A25	WP_022627580.1	60	77	0	1,368 (3,541)	1,055
CYP102A26	WP_026801194.1	61	76	0	1,352 (3,498)	1,054
CYP102D1	WP_010982013	40	57	2	776 (2,004)	1,073
Krac9955	EFH88481	42	61	2	872 (2,254)	1,080
Krac0936	EFH80345	50	67	1	1,056 (2,731)	1,074

Table S4. The distinctive derivatized mass units (sizes) for the silylated hydroxylation products of fatty acids. The sub-terminal ω-positions for hydroxylation of both saturated and unsaturated fatty acids are given.

Substrates	ω-1	ω-2	ω-3	ω-4	ω-5	ω-6
Lauric acid (C_{12})		131,331	145,317	-	-	-
Myristic acid (C_{14})	117,373	131,359	145,345	-	-	-
Palmitic acid (C_{16})	117,401	131,387	145,373	-	-	-
Margaric acid (C_{17})	117,415	131,401	145,387	-	-	-
Stearic acid (C_{18})	117,429	131,415	-	-	-	-
Oleic acid ($C_{18:1}$ cis9)	117,427	131,413	-	159,385	-	-
Linoleic acid ($C_{18:2}$ cis9)	117,425	-	-	-	-	-
Arachidonic acid ($C_{20:4}$)	117,449	-	-	-	-	-

Fig. S1. Phylogenetic tree of the holoproteins of CYP102A subfamily representatives, including CYP102A15 (*Bacillus* sp.), CYP102A170 (*Paenibacillus* sp.), CYP102A1 (*B. megaterium*), CYP102A2 (*B. subtilis*), CYP102A3 (*B. subtilis*), CYP102A5 (*B. cereus*), CYP102A7 (*B. licheniformis*), CYP102D1 (*Streptomyces avermitilis*), Krac9955 (*Ktedonobacter racemifer* DSM44963), and Krac0936 (*Ktedonobacter racemifer* DSM44963). Similarly, CYP102 members from *B. pumilus*, *B. safensis*, *B.* sp. AM 13, *B. aerophilus*, *B. stratospherericus*, *B. xiamenensis*, *B. mojavensis*, *B. halotolerans*, and *B. malacitensis* were also included. Sequences and nomenclature were obtained either from the NCBI database or P450 homepage (http://drnelson.uthsc.edu/CytochromeP450.html).

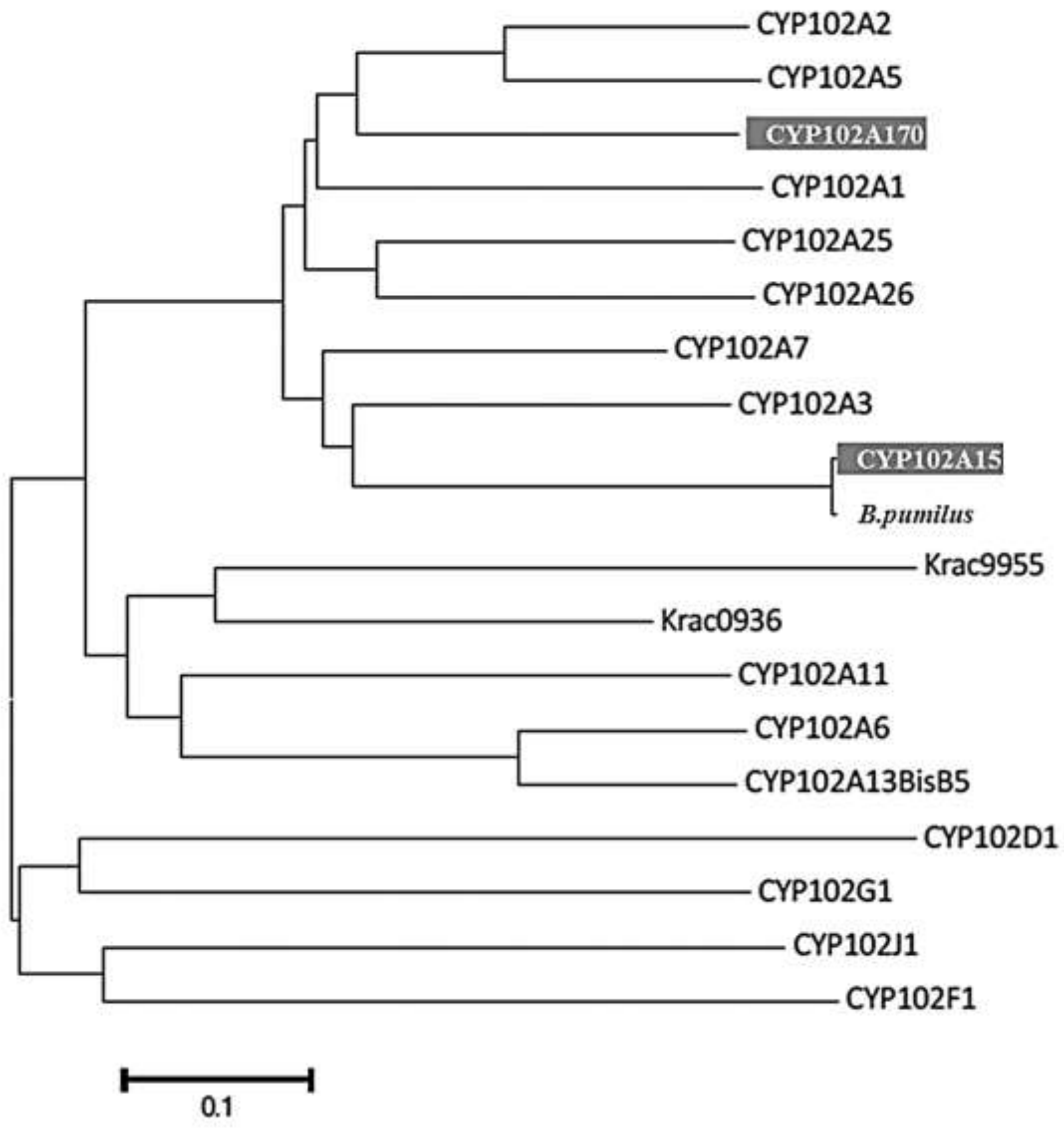

Fig. S2. SDS-PAGE analysis of protein. Where A15, M, and A170 denoted to CYP102A15 (120 kDa), molecular weight marker, and CYP1021A70 (119 kDa), respectively.

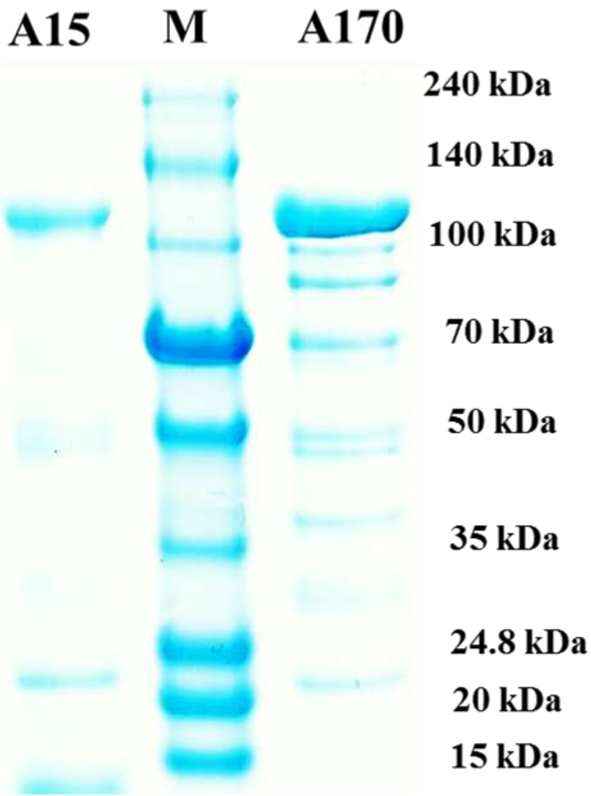

Fig. S3. Oxidized form of CYP102A15 (solid line) and lauric acid binding form (dotted line). The shift had a peak maximum at 389 nm and a minimum at 419 nm.

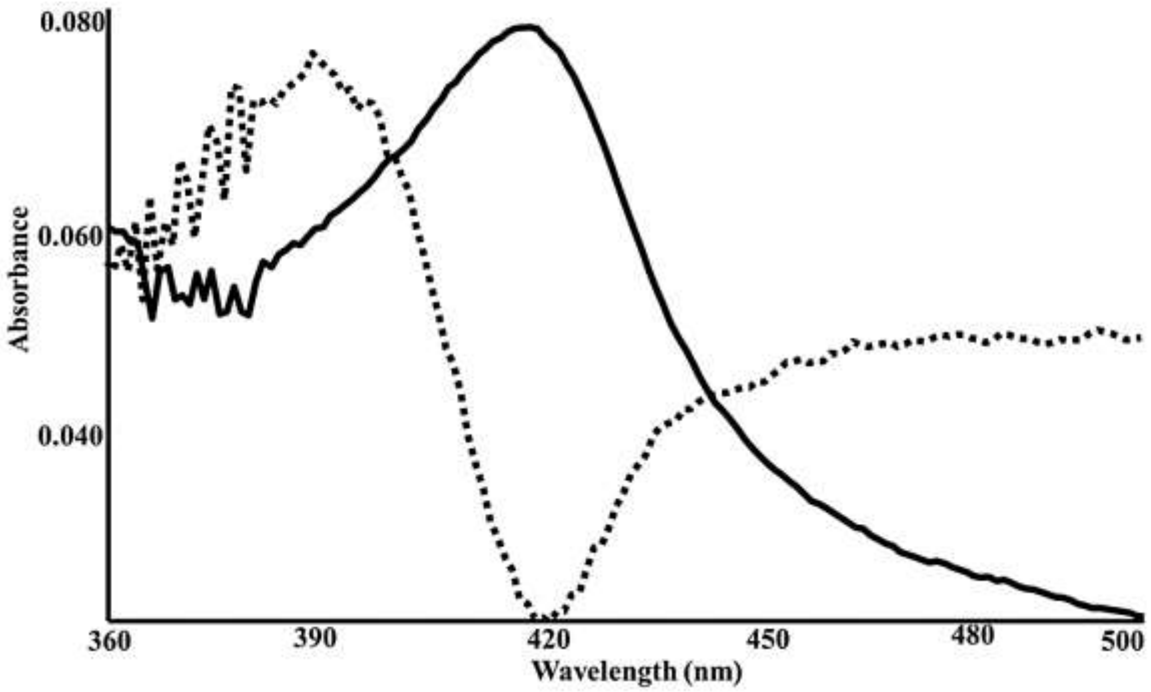

Fig. S4. The titration of the CYP102A15 with representative substrates. (**A**) The plot of absorbance change ΔA (A_{389}–A_{419}) vs. concentration of the myristic acid. Inset: difference spectra of CYP102A15 with myristic acid. The concentration of CYP102A15 was 1.5 µM. (**B**) The plot of absorbance change vs. concentration of the stearic acid. Inset: difference spectra of CYP102A15 with stearic acid. The concentration of CYP102A15 used was 2.0 µM. The titration of the CYP102A15 with representative substrates. (**C**) The plot of absorbance change ΔA (A_{389}–A_{419}) vs. concentration of the linoleic acid. Inset: difference spectra of CYP102A15 with linoleic acid. Concentration of CYP102A15 used was 2.5 µM. The shift had a peak maximum at 389 nm and a minimum at 419 nm.

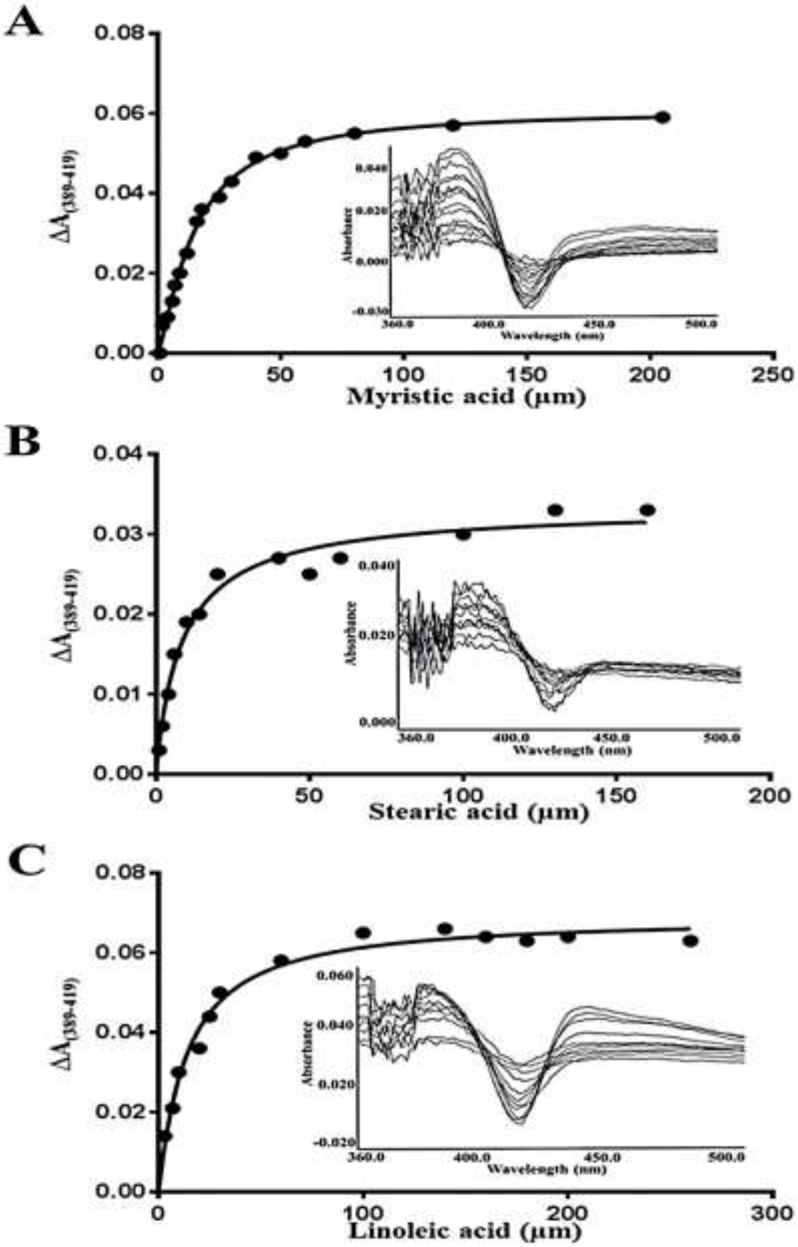

Fig. S5. (**A**) Overall GC chromatogram of subterminal ω-hydroxylation of lauric acid by CYP102A15. (**B**) GC chromatogram of ω-1 (P1). (**C**) The GC-MS spectrum of ω-1 (P1). (**D**) GC chromatogram of ω-2 (P2). (**E**) The GC-MS spectrum of ω-2 (P2). Products were confirmed by comparing with GC-MS spectra of TMS methyl-hydroxy fatty acids from lipid library (http://www.lipidlibrary.co.uk). The diagram on each MS exhibits the structure with the fragmented pattern.

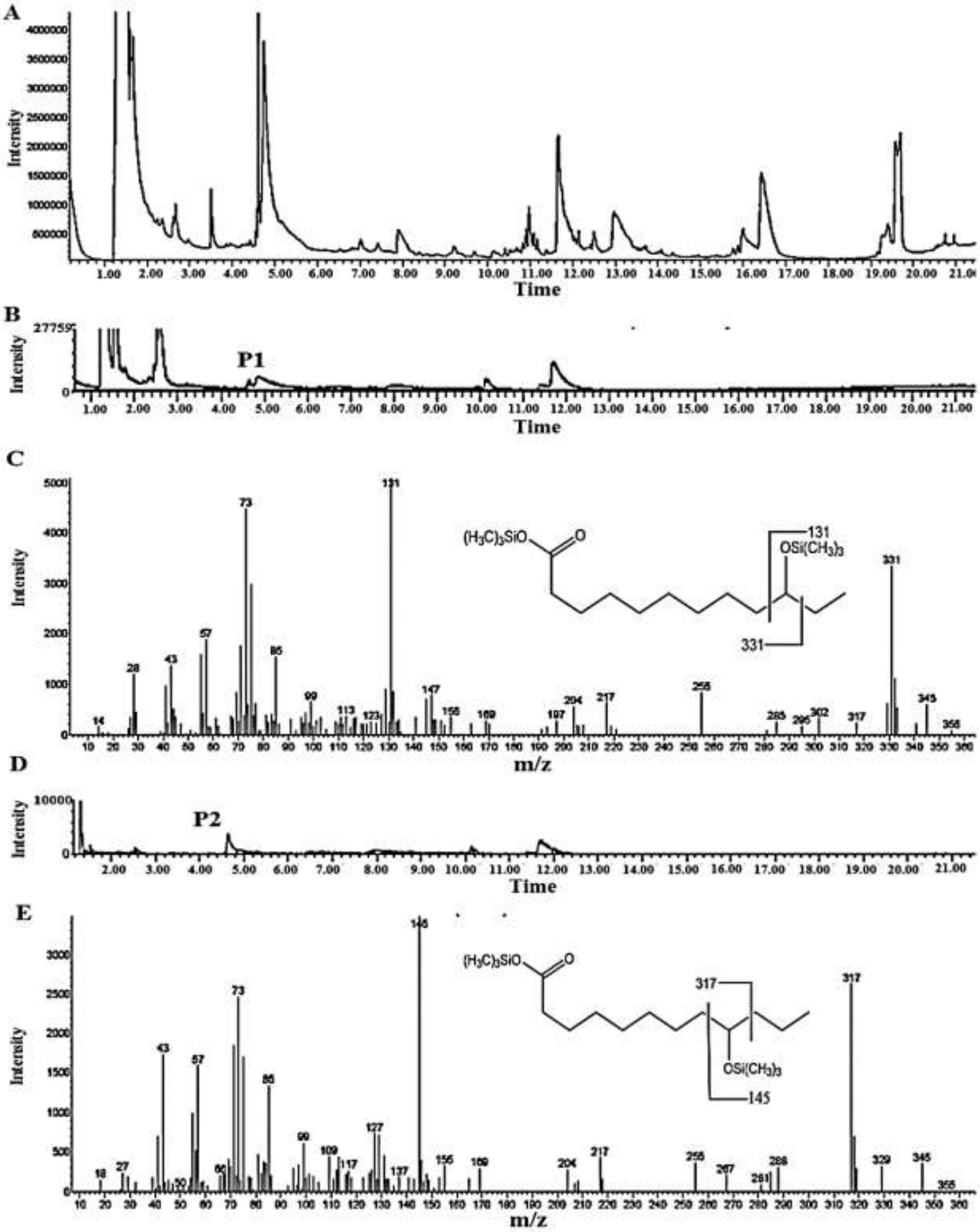

Fig. S6. (**A**) Overall GC chromatogram of subterminal ω-hydroxylation of myristic acid by CYP102A15. (**B**) GC chromatogram of ω-1. (**C**) The GC-MS spectrum of ω-1. (**D**) GC chromatogram of ω-2. (**E**) The GC-MS spectrum of ω-2. (**F**) GC chromatogram of ω-3. (**G**) The GC-MS spectrum of ω-3. The products P1, P2, and P3 were confirmed by comparing with GC-MS spectra of TMS methyl-hydroxy fatty acids from lipid library (http://www.lipidlibrary.co.uk). The diagram on each MS exhibits the structure with the fragmented pattern.

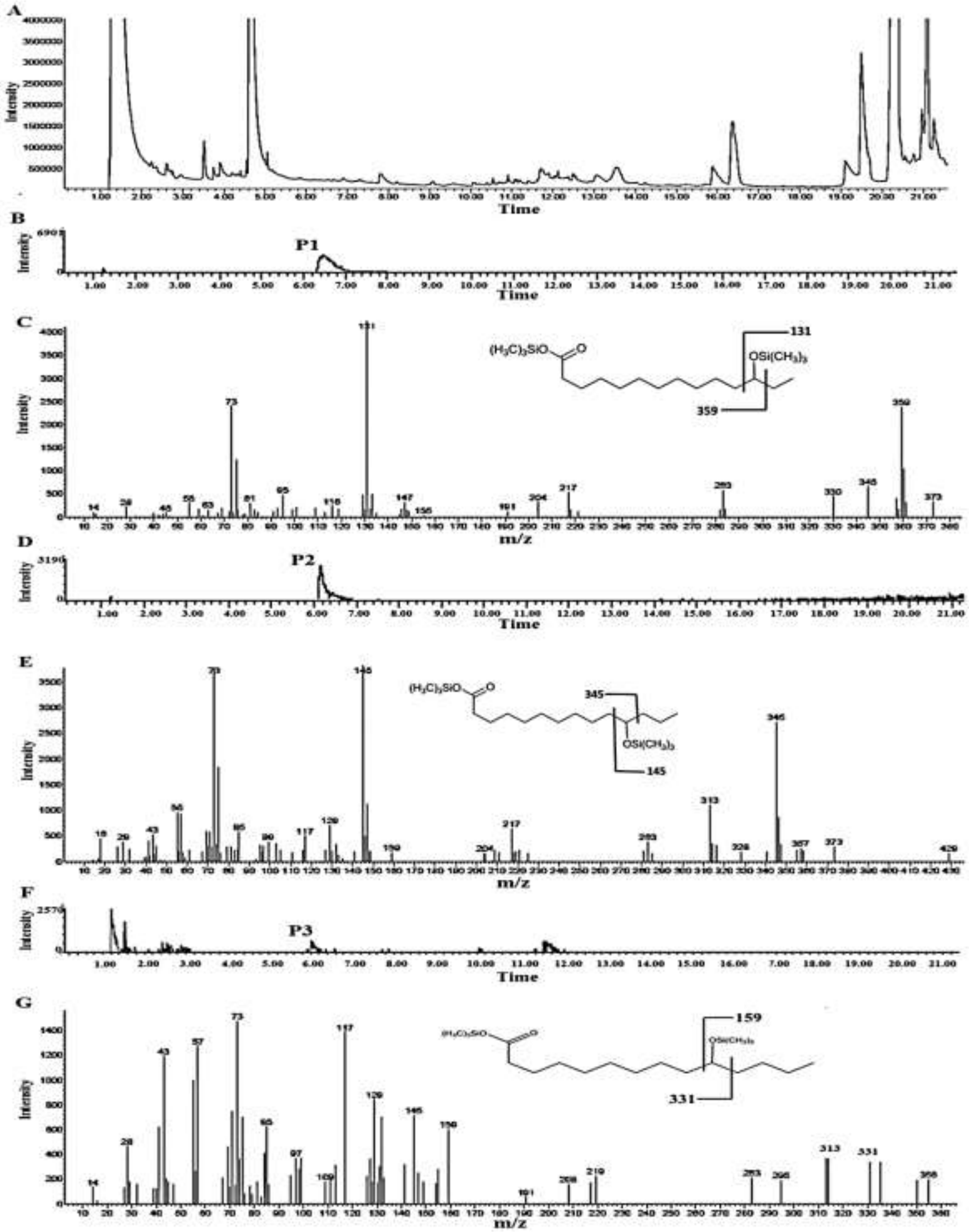

Fig. S7. (**A**) Overall GC chromatogram of subterminal ω-hydroxylation of palmitic acid by CYP102A15. (**B**) GC chromatogram of ω-1. (**C**) The GC-MS spectrum of ω-1. (**D**) GC chromatogram of ω-2. (**E**) The GC-MS spectrum of ω-2. (**F**) The highlighted GC chromatogram of ω-3. (**G**) The GC-MS spectrum of ω-3. The products P1, P2, and P3 were confirmed by comparing with GC-MS spectra of TMS methyl-hydroxy fatty acids from lipid library (http://www.lipidlibrary.co.uk). The diagram on each MS exhibits the structure with the fragmented pattern.

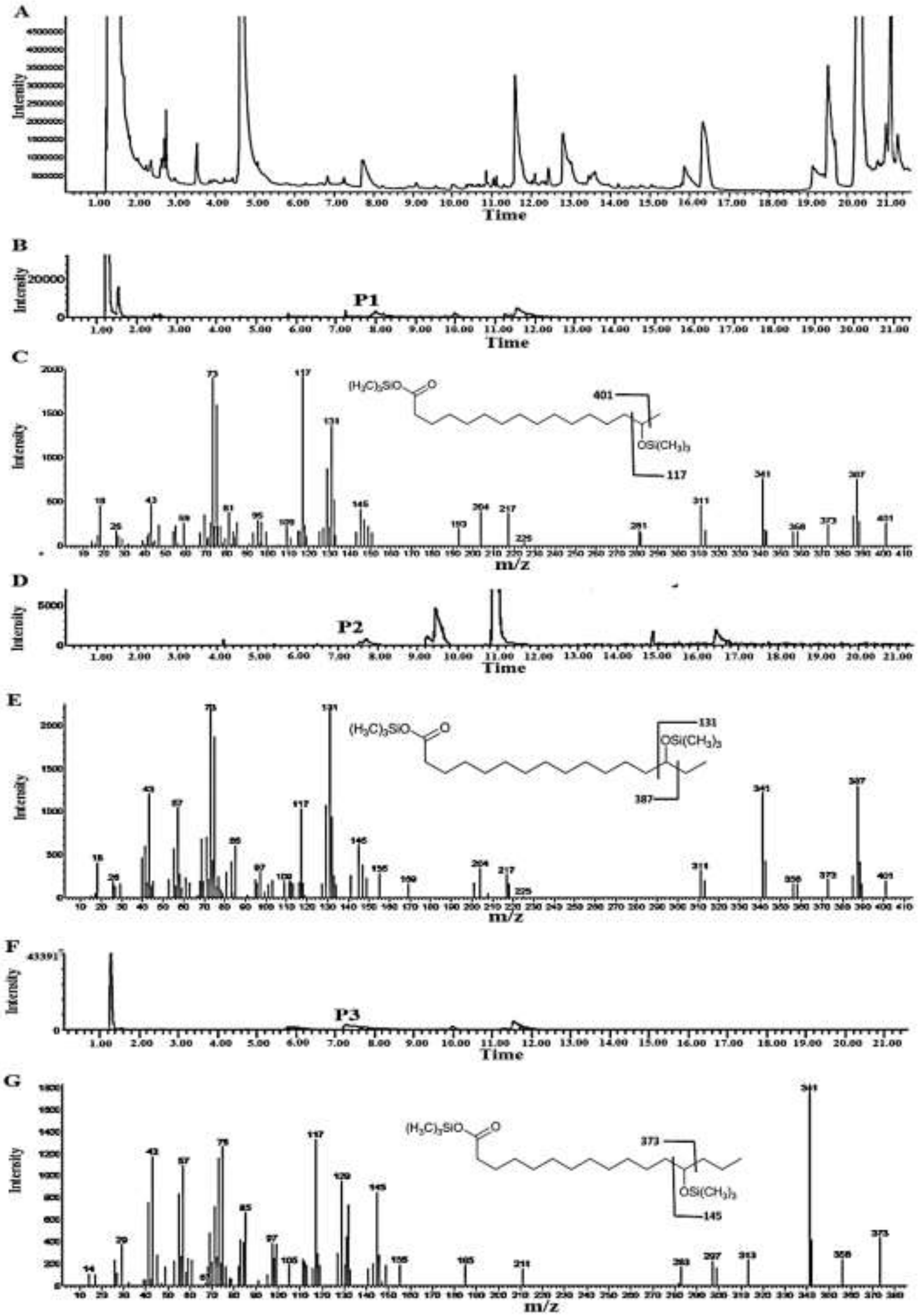

Fig. S8. (**A**) Overall GC chromatogram of subterminal ω-hydroxylation of margaric acid by CYP102A15. (**B**) The GC-MS spectrum of ω-1 (P1) hydroxylated products. (**C**) The GC-MS spectrum of ω-2 (P2) hydroxylated products.

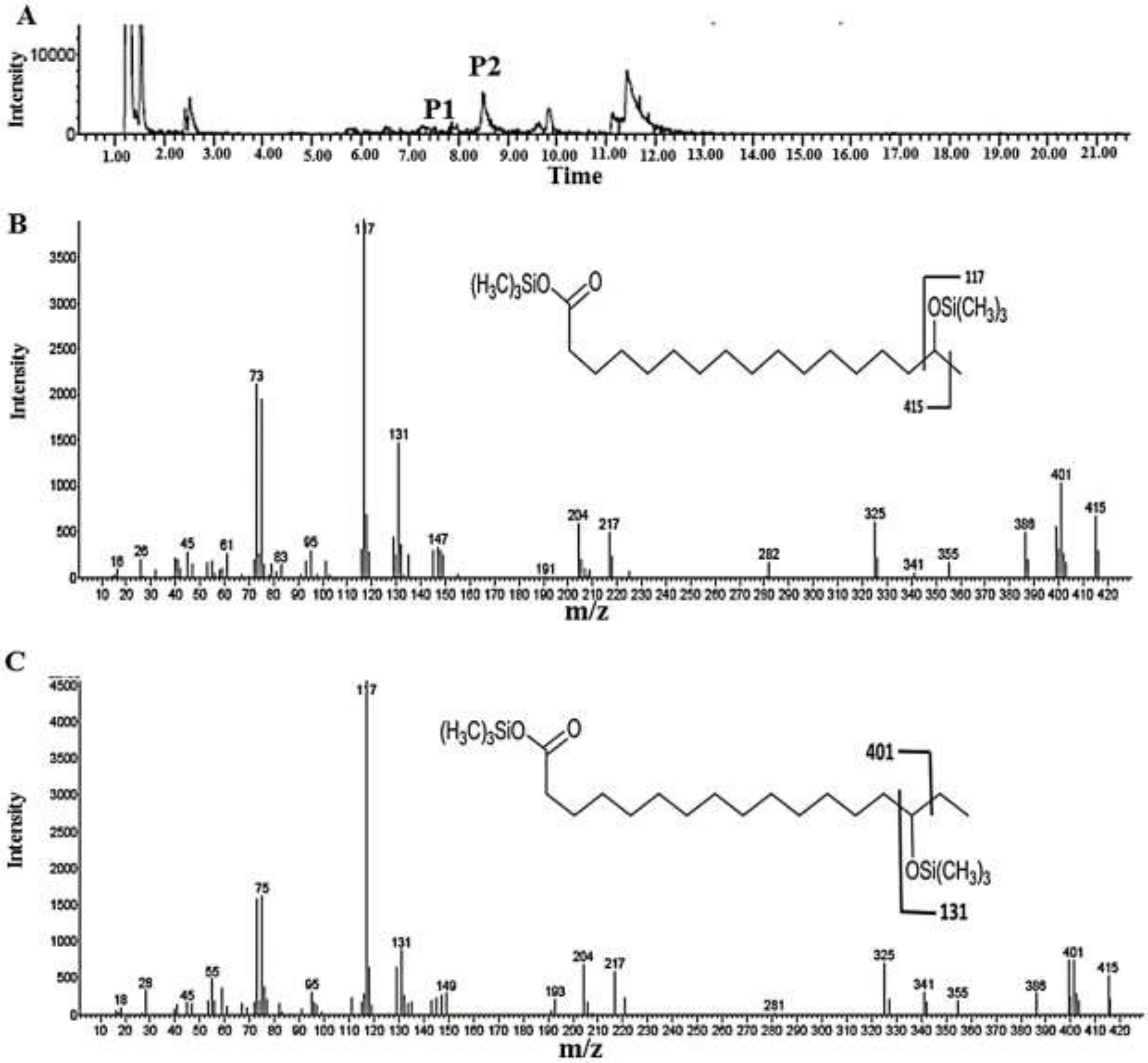

Fig. S9. (**A**) Overall GC chromatogram of subterminal ω-hydroxylation of oleic acid by CYP102A15. (**B**) GC chromatogram of ω-1 (P1) and ω-2 (P2) hydroxylated products of oleic acid. (**C**) The GC-MS spectrum of ω-1 (P1) hydroxylated products of oleic acid. (**D**) The GC-MS spectrum of ω-2 (P2) hydroxylated products of oleic acid. The products P1 and P2 were confirmed by comparing with GC-MS spectra of TMS methyl-hydroxy fatty acids from lipid library (http://www.lipidlibrary.co.uk). The diagram on each MS exhibits the structure with the fragmented pattern.

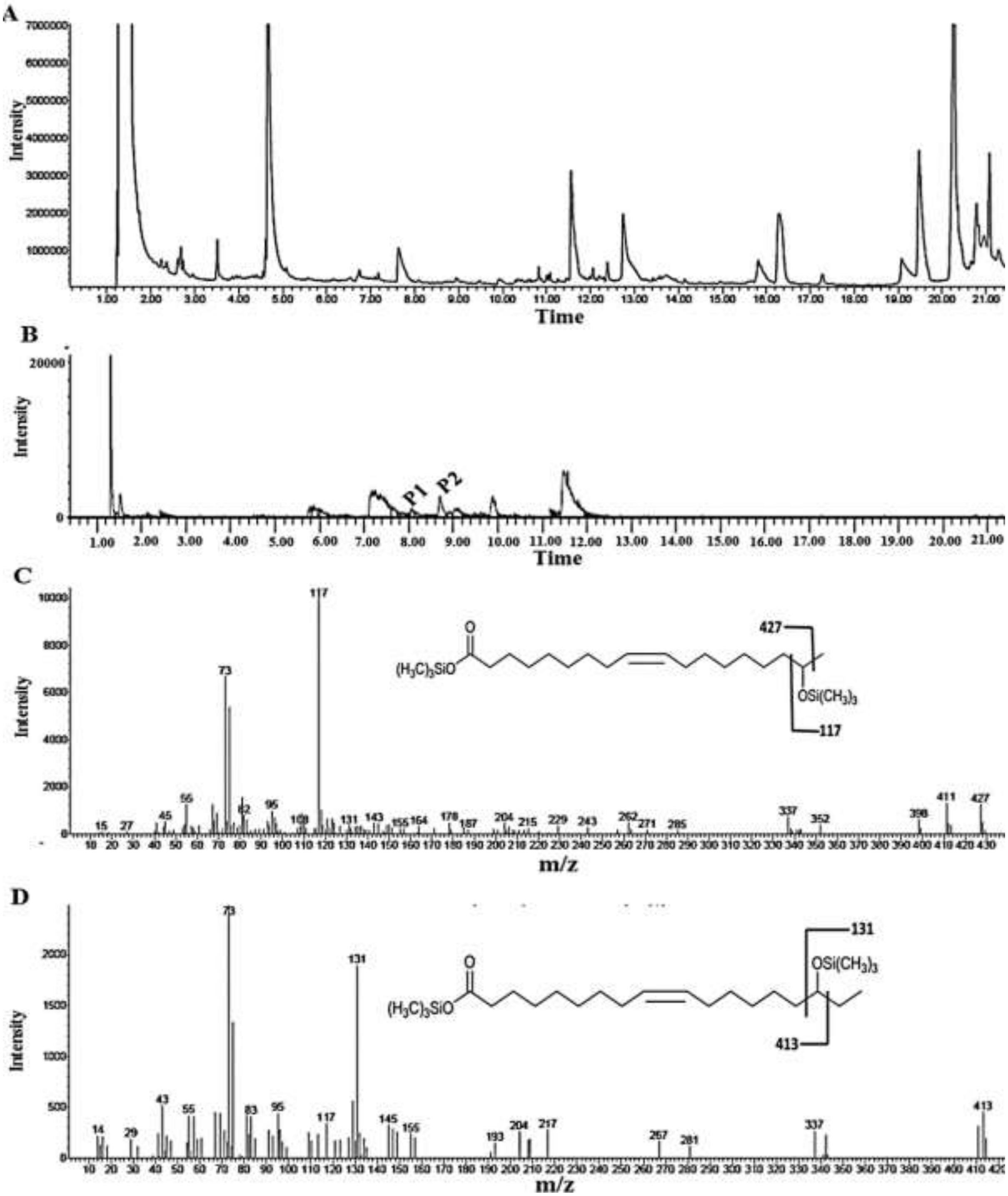

Fig. S10. (**A**) Overall GC chromatogram of subterminal ω-hydroxylation of linoleic acid by CYP102A15. (**B**) The GC chromatogram of ω-1 (P) hydroxylated products of linoleic acid. (**C**) The GC-MS spectrum of ω-1 (P) hydroxylated products of lionoleic acid.

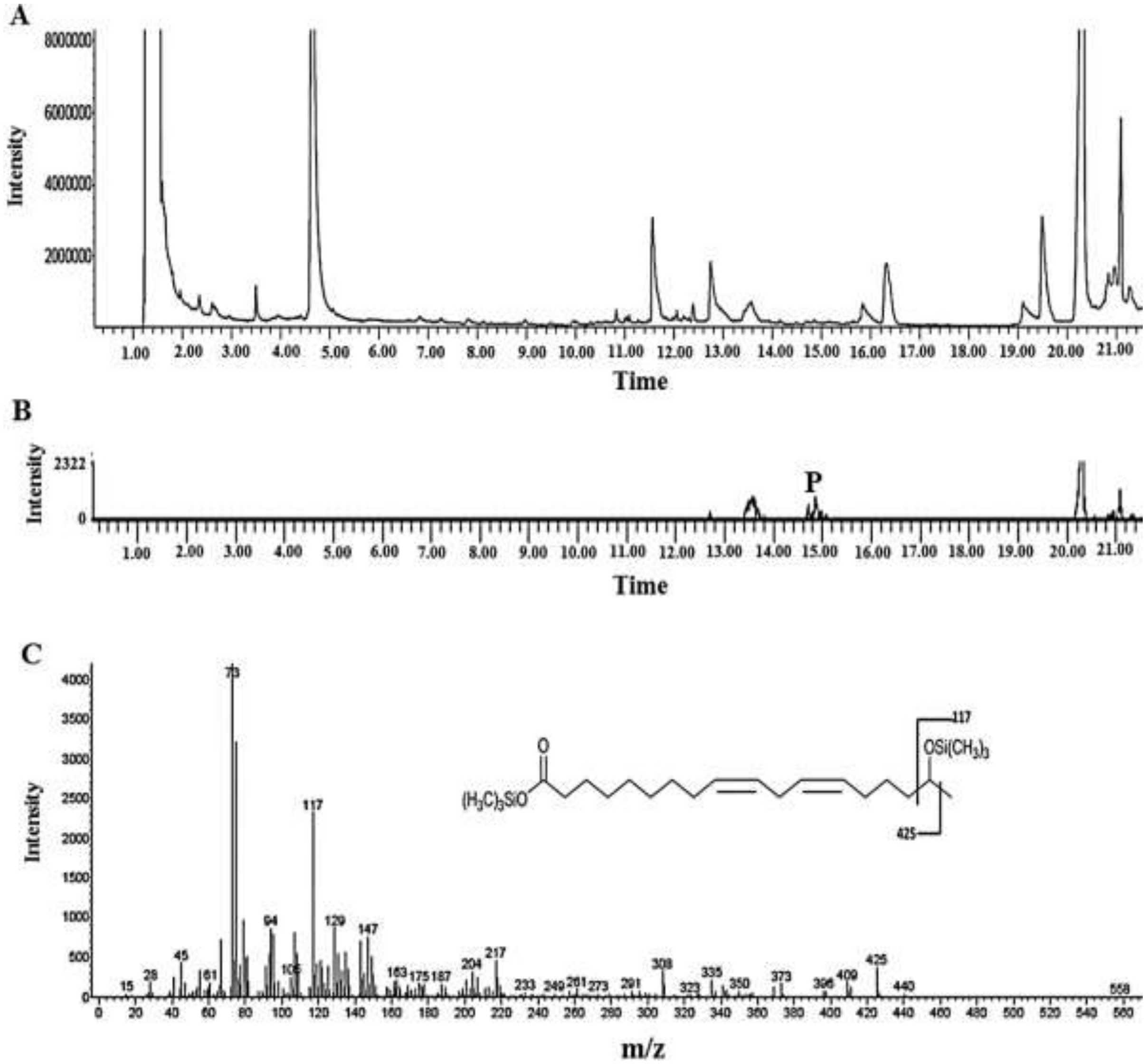

Fig. S11. The titration of the CYP102A170 with representative substrates. (**A**) The plot of absorbance change ΔA (A_{390}–A_{420}) vs. concentration of the palmitic acid. Inset: difference spectra of CYP102A170 with palmitic acid. (**B**) The plot of absorbance change vs. concentration of the oleic acid. Inset: difference spectra of CYP102A170 with oleic acid. (**C**) The plot of absorbance change vs. concentration of the stearic acid. Inset: difference spectra of CYP102A170 with stearic acid. (**D**) The plot of absorbance change vs. concentration of the arachidonic acid. Inset: difference spectra of CYP102A170 with arachidonic acid. The concentration of CYP102A170 used was 1.5 μM.

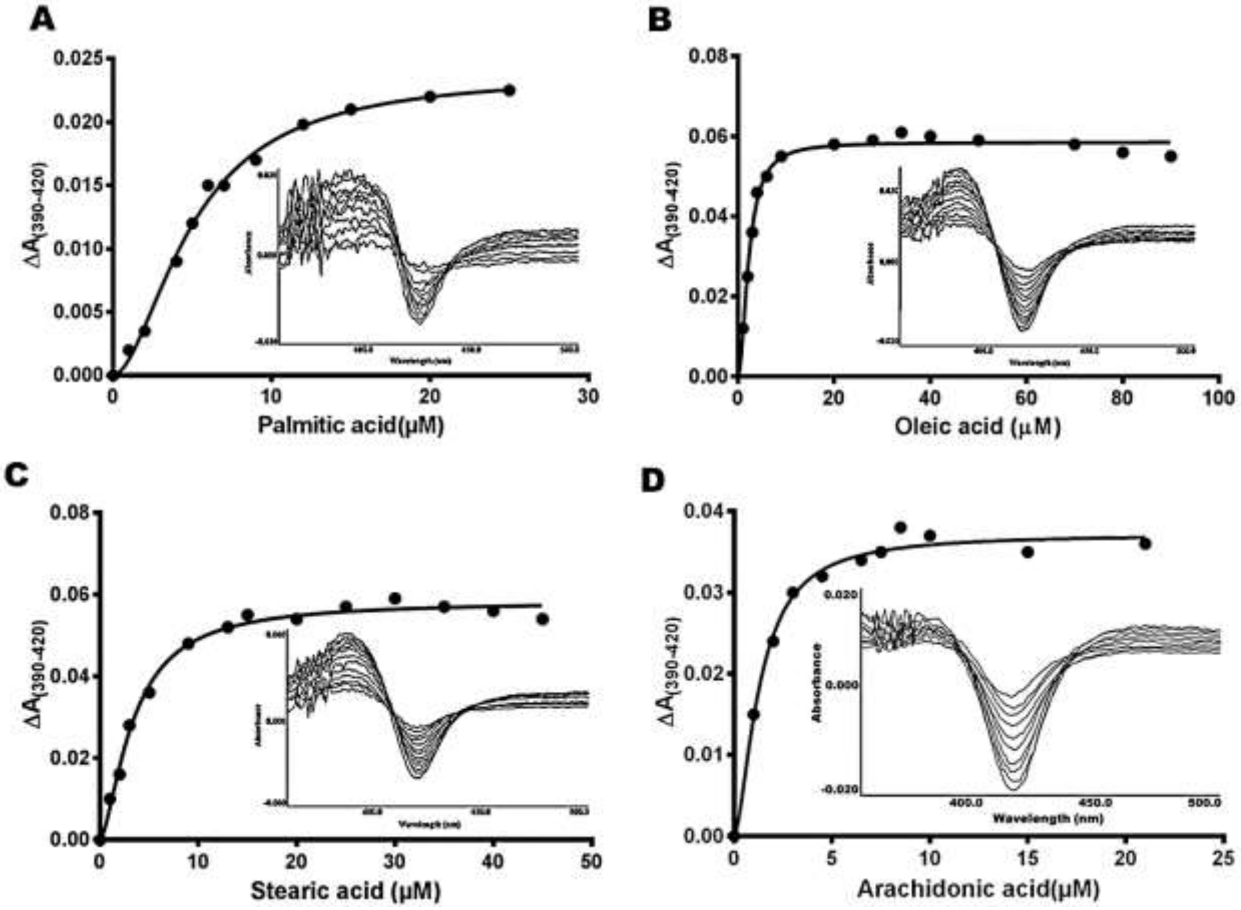

Fig. S12. (**A**) GC chromatogram of subterminal ω-hydroxylation of stearic acid by CYP102A15. Both the products P1 and P2 were confirmed by comparing with GC-MS spectra of TMS methyl-hydroxy fatty acids from lipid library (http://www.lipidlibrary.co.uk). The diagram on each MS exhibits the structure with the fragmented pattern. (**B**) The GC-MS spectrum of ω-1 (P1) hydroxylated products. (**C**) The GC-MS spectrum of ω-2 (P2) hydroxylated products.

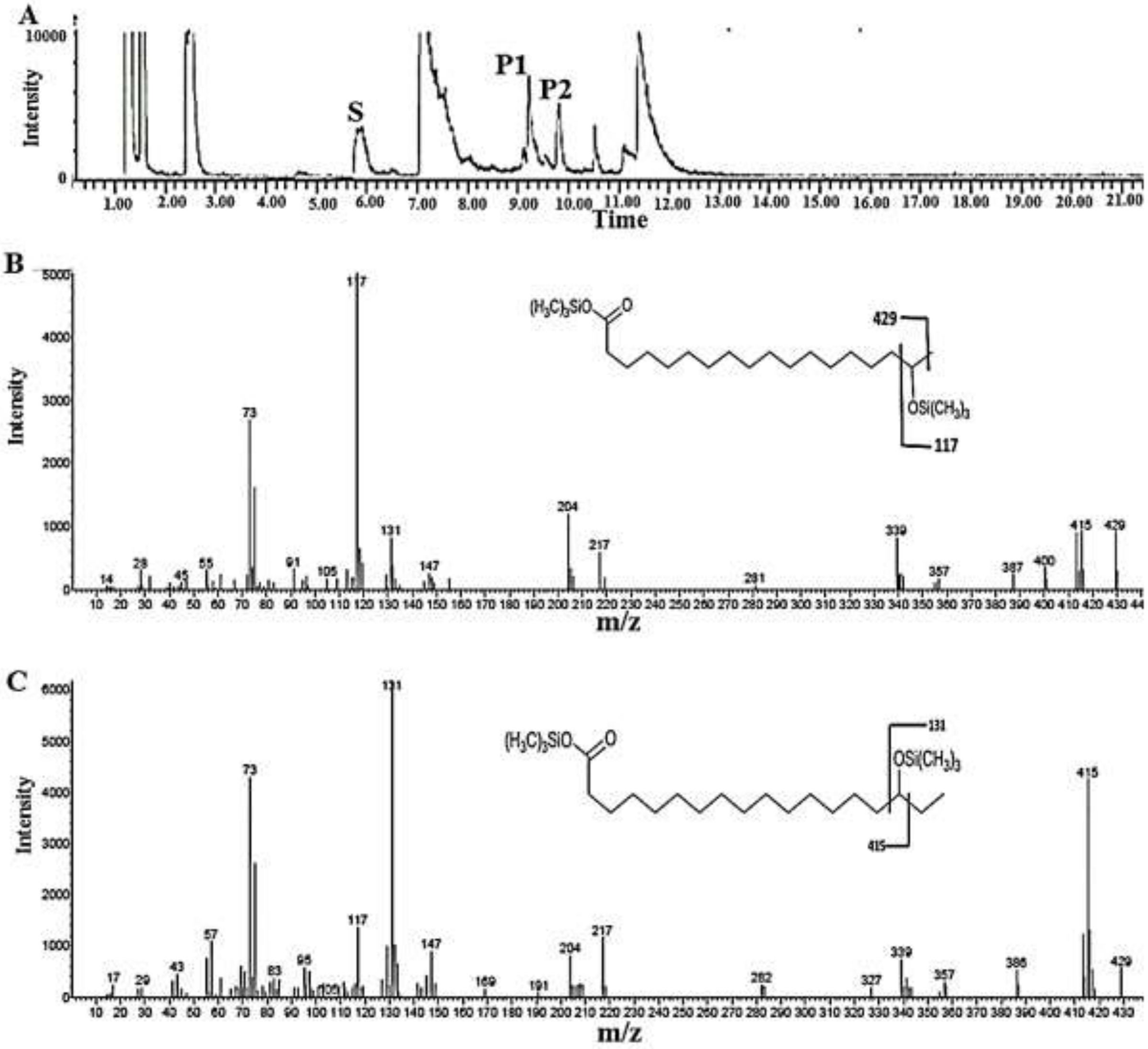

Fig. S13. GC chromatogram and mass analysis of subterminal ω-hydroxylation of myristic acid by CYP102A170. (**A**) The GC spectrum of ω-1 (P1) hydroxylated products. (**B**) The GC-MS spectrum of ω-1 (P1) hydroxylated products. (**C**) The GC spectrum of ω-2 (P2) hydroxylated products. (**D**) The GC-MS spectrum of ω-2 (P2) hydroxylated products. Both the products P1 and P2 were confirmed by comparing with GC-MS spectra of TMS methyl-hydroxy fatty acids from lipid library (http://www.lipidlibrary.co.uk). The diagram on each MS exhibits the structure with the fragmented pattern.

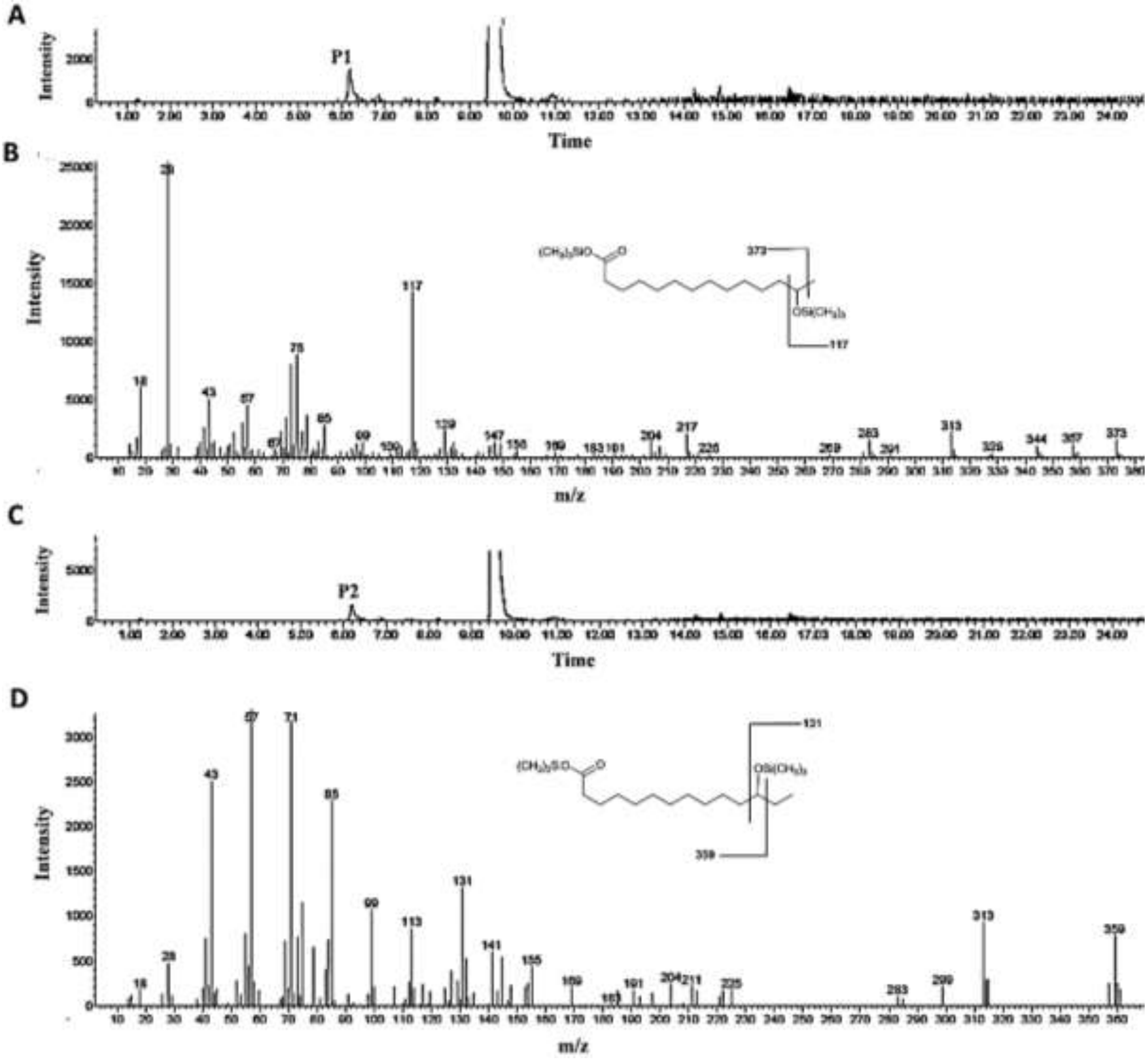

Fig. S14. GC chromatogram and mass analysis of subterminal ω-hydroxylation of palmitic acid by CYP102A170. (**A**) The GC spectrum of ω-1 (P1), ω-2 (P2), and ω-3 (P3) hydroxylated products of palmitic acid. (**B**) The GC-MS spectrum of ω-1 (P1). (**C**) The GC-MS spectrum of ω-2 (P2). (**D**) The GC-MS spectrum of ω-3 (P3). All the products P1, P2, and P3 were confirmed by comparing with GC-MS spectra of TMS methyl-hydroxy fatty acids from lipid library (http://www.lipidlibrary.co.uk). The diagram on each MS exhibits the structure with the fragmented pattern.

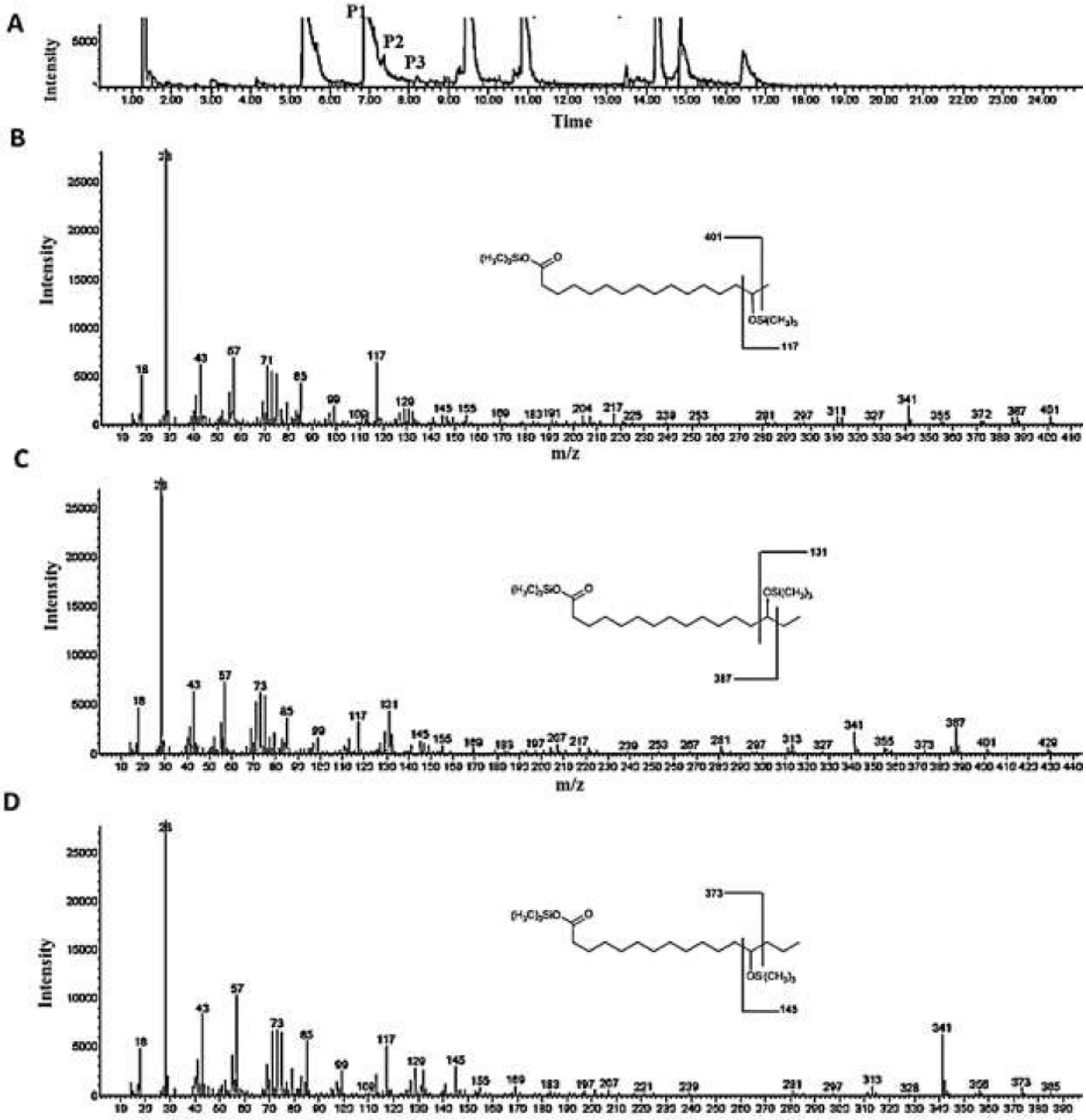

Fig. S15. GC chromatogram and mass analysis of subterminal ω-hydroxylation of stearic acid by CYP102A170. (**A**) The GC spectrum of ω-1 (P1) and ω-2 (P2) hydroxylated products of stearic acid. (**B**) The GC-MS spectrum of ω-1 (P1) hydroxylated products of stearic acid. (**C**) The GC-MS spectrum of ω-2 (P2). Both the products P1 and P2 were confirmed by comparing with GC-MS spectra of TMS methyl-hydroxy fatty acids from lipid library (http://www.lipidlibrary.co.uk). The diagram on each MS exhibits the structure with the fragmented pattern.

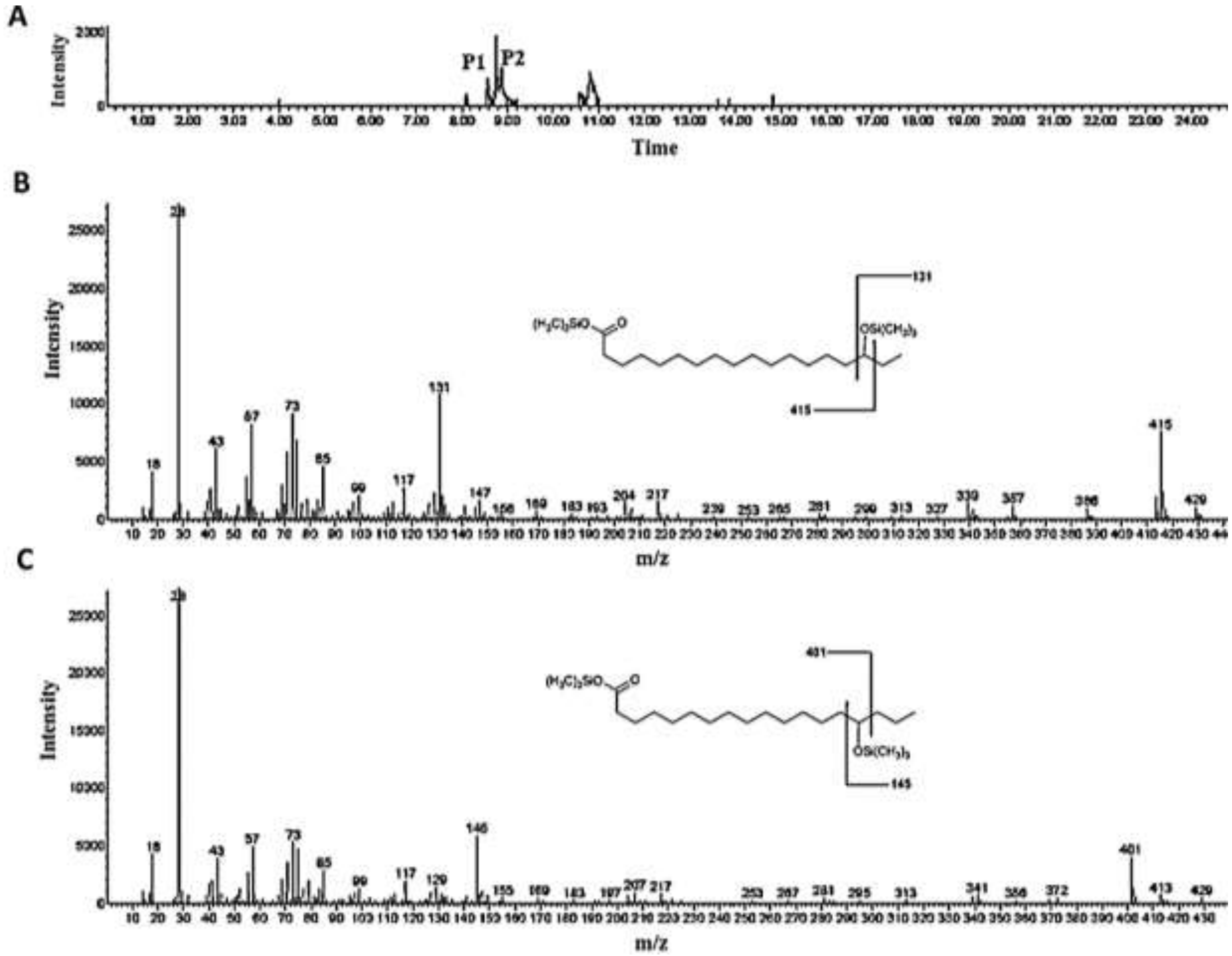

Fig. S16. GC chromatogram and mass analysis of subterminal ω-hydroxylation of margaric acid by CYP102A170. (**A**) The GC spectrum of ω-1 (P1). (**B**) The GC-MS spectrum of ω-1 (P1). (**C**) The GC spectrum of ω-2 (P2). (**D**) The GC-MS spectrum of ω-2 (P2). All the products P1 and P2 were confirmed by comparing with GC-MS spectra of TMS methyl-hydroxy fatty acids from lipid library (http://www.lipidlibrary.co.uk). The diagram on each MS exhibits the structure with the fragmented pattern.

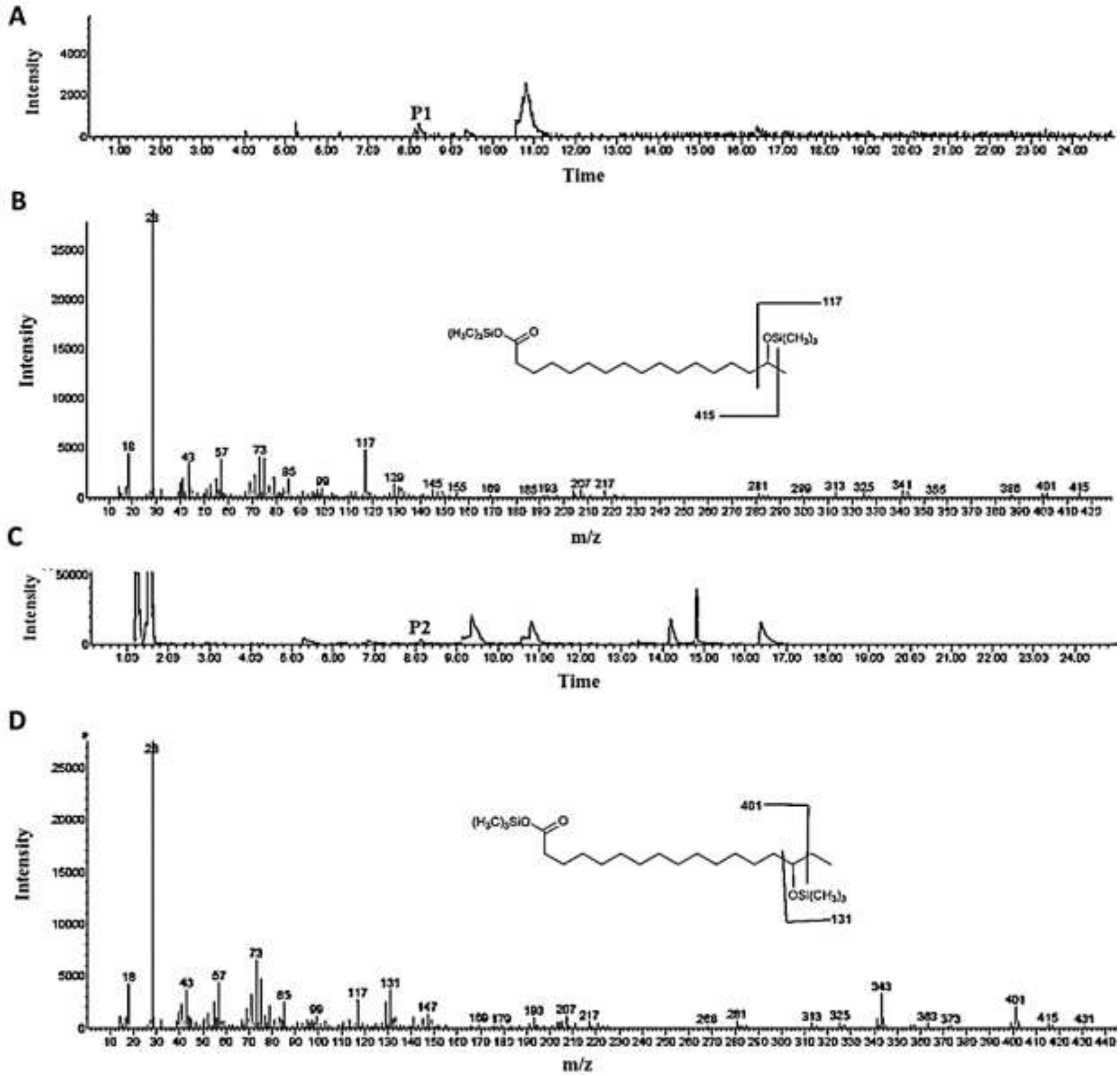

Fig. S17. GC chromatogram and mass analysis of subterminal ω-hydroxylation of oleic acid by CYP102A170. (**A**) The GC spectrum of ω-1 (P1) and ω-2 (P2) hydroxylated products. (**B**) The GC-MS spectrum of ω-1 (P1). (**C**) The GC-MS spectrum of ω-2 (P2). Both the products P1 and P2 were confirmed by comparing with GC-MS spectra of TMS methyl-hydroxy fatty acids from lipid library (http://www.lipidlibrary.co.uk). The diagram on each MS exhibits the structure with the fragmented pattern.

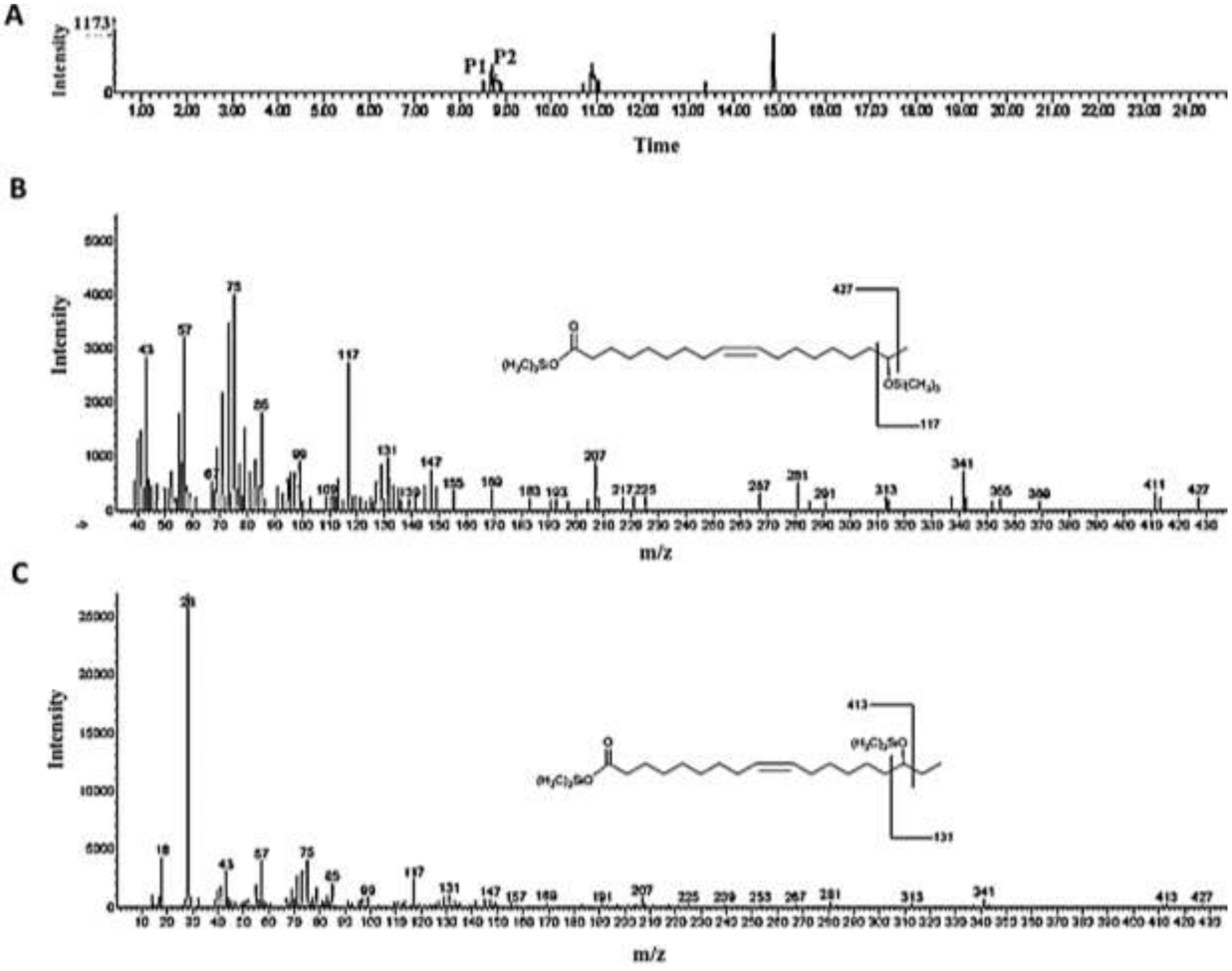

Fig. S18. GC chromatogram and mass analysis of subterminal ω-hydroxylation of linoleic acid by CYP102A170. (**A**) The GC spectrum of ω-1 (P) hydroxylated products. (**B**) The GC-MS spectrum of ω-1 (P) hydroxylated products. The product P was confirmed by comparing with GC-MS spectra of TMS methyl-hydroxy fatty acids from lipid library (http://www.lipidlibrary.co.uk). The diagram on each MS exhibits the structure with the fragmented pattern.

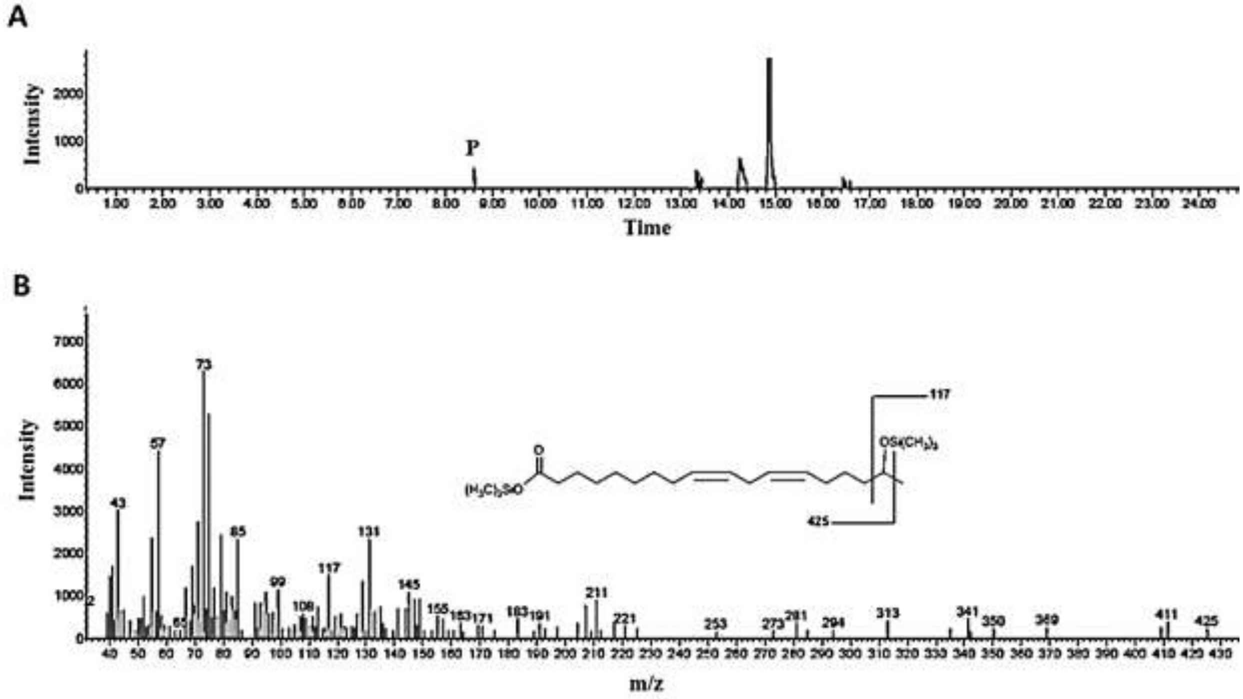

Fig. S19. GC chromatogram and mass analysis of subterminal ω-hydroxylation of eicosapentaenoic acid by CYP102A170. (**A**) The GC spectrum of ω-1 (P) hydroxylated products. (**B**) The GC-MS spectrum of ω-1 (P) hydroxylated products. The product P was confirmed by comparing with GC-MS spectra of TMS methyl-hydroxy fatty acids from lipid library (http://www.lipidlibrary.co.uk). The diagram on each MS exhibits the structure with the fragmented pattern.

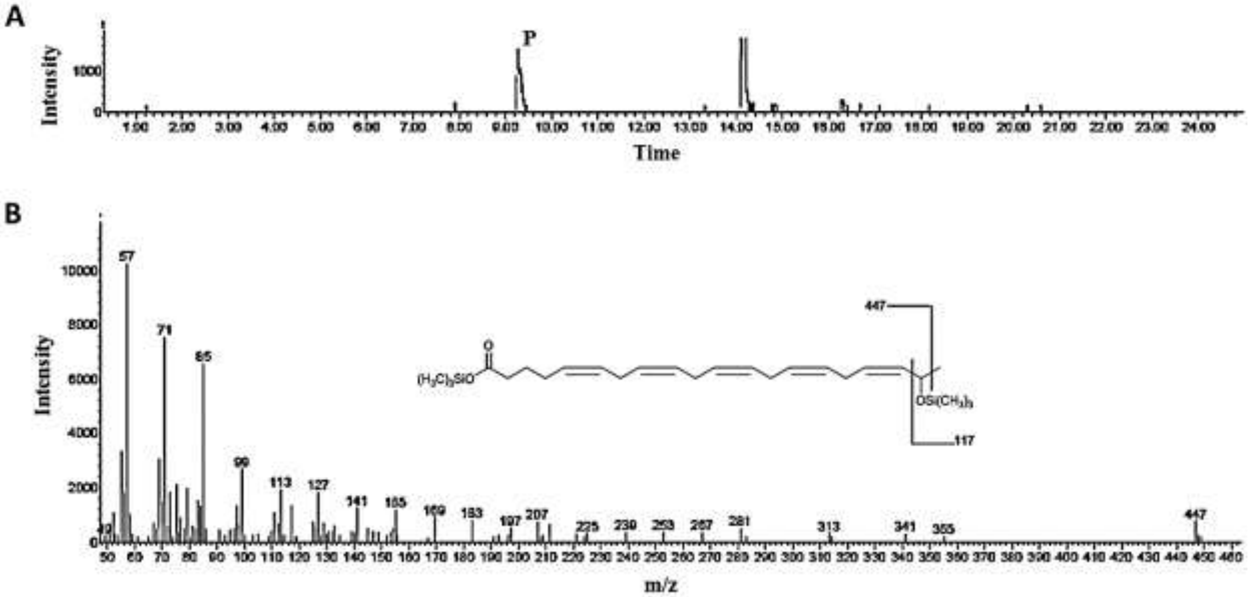

Supplement: Supplementary file 1 [file JMB-30-5-777-supple.pdf]
